# Nanoscale Cross-Sectional Characterization of Thin Layers in Material Assemblies

**DOI:** 10.3390/nano15110840

**Published:** 2025-05-30

**Authors:** Frédéric Addiego, Rutuja Bhusari, Julien Bardon, Sascha Scholzen, Zainhia Kaidi

**Affiliations:** 1 Structural Composites Unit, Luxembourg Institute of Science and Technology, 5 Avenue des Hauts-Fourneaux, L-4362 Esch-Belval Esch-sur-Alzette, Luxembourgjulien.bardon@list.lu (J.B.); 2 Circuit Foil Luxembourg, 6 Salzbaach, L-9559 Wiltz, Luxembourg; sascha.scholzen@circuitfoil.com (S.S.); zainhia.kaidi@circuitfoil.com (Z.K.)

**Keywords:** material assembly, adhesive, release layer, nanoscale, structure, characterization

## Abstract

Thin-film assemblies containing an adhesion layer (AdL) or a release layer (RL) with nanoscale thickness are widely used in semiconductors, electrical circuit boards, optical and optoelectronic devices, photodiodes, and photonics applications. Current environmental concerns and technological demands necessitate continuous advancements in these nano-AdLs and nano-RLs in terms of formulation, design, functionality, and durability. Developing these nano-layers relies on understanding their structural properties, which is challenging because only characterization tools with nanoscale or sub-nanoscale lateral resolution can be employed. The aim of this review is to provide an overview of the current techniques and methods available for characterizing the structural properties of nano-layers in cross-section. Emphasis is placed on sample preparation methods, the fundamental principles, advantages, and limitations of various techniques, and examples from the existing literature. First, selecting the appropriate characterization technique depends on the required lateral resolution—it must be finer than the size of the structural feature of interest. A high lateral resolution relative to this structural feature translates to more accurate characterization, enabling effective profiling and mapping analysis. Subsequently, it is important to optimize sample preparation regarding shape, dimensions, and surface roughness, while minimizing artifacts. Combining techniques that offer complementary structural information—such as morphological, chemical, and nanomechanical data—is recommended to gain a comprehensive understanding of the nano-layer’s structure and properties. This is especially important when utilizing 3D characterization methods. It is worth noting that few examples of cross-sectional analysis for nano-AdLs and nano-RLs are available in the literature, highlighting the need for further nanoscale investigations. This review aims to serve as a practical guide for scientists, helping them identify suitable characterization procedures based on the specific structural information they seek to obtain.

## 1. Introduction

An adhesion layer (AdL) can be defined as a material with a controlled thickness that permanently bonds two bodies (also called adherends) of different chemical compositions. This bonding involves the mechanical strength or energy required for separation, typically ranging between 100 and 1000 J/m^2^ [1]. In the current literature and practice, the term “adhesion” can be described by words such as bonding, sticking, or gluing. Similarly, a “layer” (or interlayer) can be referred to as an agent, promoter, compound, material, coating, joint, film, or foil. The adhesion layer may also be simply called an adhesive or glue. Throughout this manuscript, the terms AdL and adhesion are used for simplicity, acknowledging that the focus is on material assemblies characterized by multiple layers of different materials. It is important to note that the material used as an AdL must exhibit significant physico-chemical interactions with the two adherends. Conversely, a release layer (RL), also known as a sacrificial, separation, disassembling, or debonding layer, is a material with controlled thickness that temporarily bonds two bodies of different chemical compositions. Upon applying an external stimulus—such as thermo-mechanical stress, laser ablation, or chemical dissolution [2,3]—the two bodies are separated. The energy required to separate the assembly via an RL is generally below 100 J/m^2^ [3,4,5,6]. Typically, the RL has limited physico-chemical interactions with the adherends, exhibits peeling properties [7], or has an irregular atomic structure, such as a liquid state [8].

For both AdL and RL, the layer’s thickness—ranging from nanometers to millimeters depending on the assembly design—significantly influences the adhesion strength [3,6,8,9,10,11,12,13]. However, the impact of layer thickness on adhesion is subject to ongoing debate. In some studies, layers with micron-to-millimeter thickness show that thinner layers produce higher mechanical strength during separation. This is attributed to reduced stress localization at the interface and fewer defects within the layer, causing failure paths to propagate through the middle of the layer, allowing it to absorb more strain and maintain higher strength [12]. Conversely, thicker layers tend to fail at the layer–body interface, reducing their contribution to overall strength. Other research indicates that the improved mechanical strength of thin layers may stem from their lower yield stress due to high internal stresses, leading to plastic deformation at lower applied stresses and greater energy dissipation [6]. With nanometer-scale layers, bonding strength depends heavily on molecular or atomic contact, and is affected by surface roughness, surface conformity, and physico-chemical interactions. The relationship between layer thickness and adhesion strength is complex and not clearly defined in the literature. Reducing the thickness of an AdL is currently a challenging, resource-intensive approach, driven by sustainability and technological considerations. Similarly, minimizing RL thickness is crucial for minimizing alterations to surface morphology after separation [2,14].

This review focuses on thin layers—nano-AdLs and nano-RLs—ranging from about 1 nanometer to a few hundred nanometers. Analyzing their structural properties presents significant challenges and requires advanced sample preparation and characterization techniques. A review of the most recent 150 documents related to nano-AdLs, retrieved from the SciFinder database [15], reveals that these layers are mainly associated with ceramics, semiconductors (silicon, silica), and metals (copper, gold, titanium, silver), with silicon being particularly prominent. Silicon can function either as an adherend [16] or as the adhesive itself [17]. Similarly, metals like copper may serve as adherends [18] or as an AdL formed through diffusion bonding [19]. A parallel analysis of the latest 150 documents concerning nano-RLs shows that these layers are related predominantly to ceramics, semiconductors (silicon, silica, carbon, graphene), metals (gold, aluminum, chromium, copper, titanium, silver), and polymers (e.g., poly(methyl methacrylate)). Silicon, silica, and gold are particularly noteworthy. Silicon can be part of the assembly substrate [20] or the RL [21], while gold may function as either an adherend [22] or an RL [23]. These nano-AdLs and nano-RLs are integrated into thin-film technologies used across semiconductors [16,24,25,26,27,28,29,30], electrical circuit boards [8,31,32,33,34], optical and optoelectronic devices [21,22,35,36], photodiodes [37], photonics applications [38], and power modules [39]. Additionally, nano-AdLs are employed in various electronic applications beyond thin-film technologies, including battery electrode bonding systems [40], interconnection systems [41,42], and heat dissipation systems for LEDs [43], with ongoing efforts to develop universal nano-AdL solutions for diverse applications [44,45].

Recent advancements in the field of nano-AdLs and nano-RLs involve the formulation, design, and development of novel layers to address various challenges. These include the following, among other factors:

(i) New environmental regulations, such as the Registration, Evaluation, Authorization, and Restriction of Chemicals (REACH) and the Restriction of Hazardous Substances (RoHS) Directive in Europe [3,46].

(ii) Miniaturization driven by 5G, artificial intelligence, and the Internet of Things [2,8,28,34].

(iii) The introduction of flexible substrates [33,47] and organic materials in electronic applications [25,34].

(iv) New layer functionalities, such as self-healing adhesives [48,49] or diffusion suppression between assembly components in the case of RL [34].

(v) Multifunctionality, including for example the development of adhesives that are both thermally conductive and anti-aging [43], and adhesives that are both flame-retardant and anti-aging [50].

(vi) Room-temperature adhesion to prevent the structural modification of substrates [29,32,41].

To determine whether these new nano-AdLs and nano-RLs effectively meet their targeted functionalities and durability requirements, it is crucial to characterize their structural properties at the atomic or molecular level. This necessitates the use of characterization tools with nanoscale or sub-nanoscale resolution, capable of uncovering the hidden intricacies at the nanometer scale.

Building on previous work concerning nanoparticle characterization [51], this review aims to outline the most advanced techniques available for analyzing the structural properties of nano-AdLs and nano-RLs. The focus is on methods that enable the assessment of local structural features through mapping at at least the nanoscale. The first section briefly discusses sample preparation methods, as this step significantly influences the analysis of nano-AdLs and nano-RLs within material assemblies. Then, the principles, types of structural information obtained, and limitations of each relevant technique will be summarized, supported by recent literature examples, including investigations of nano-AdLs and nano-RLs when available. Some emerging techniques are also identified and briefly reviewed at the end of the manuscript. This review serves as a practical guide for scientists aiming to identify the structural fingerprint of the nano-AdL or nano-RL they are developing or studying. The techniques are categorized in Table 1 and Table 2 into those providing 2D information and those offering 3D insights. When the structural feature of interest is homogeneously distributed throughout the nanolayer, with no significant variation in chemical composition, shape, or size, a 2D investigation based on a few cross-sections may provide representative information. In contrast, if such uniformity is absent, 2D investigations should be conducted on multiple cross-sections to capture the full range of variability, or a 3D investigation should be undertaken to obtain more representative data.

## 2. Sample Preparation Methods

**Figure 1:**
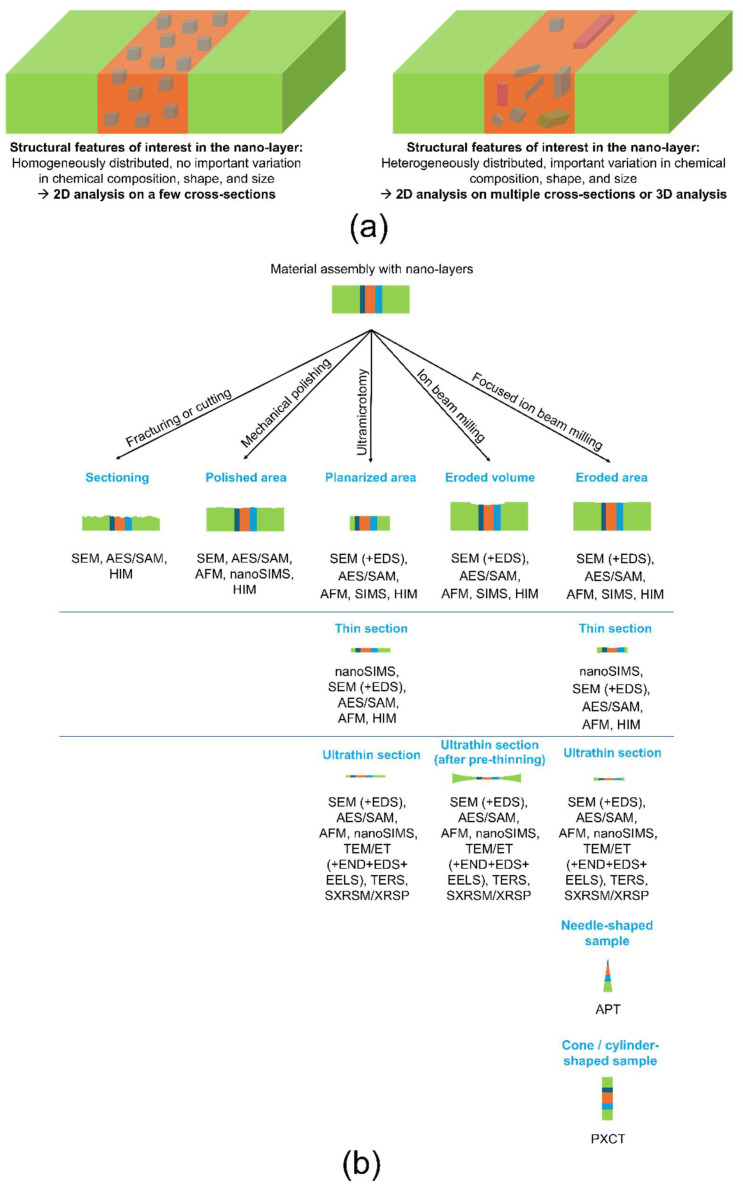
(**a**) Potential variability in structural features of interest and corresponding characterization strategies, and (**b**) summary of methods for preparing cross-sections of material assemblies (the light green layers represent thick outer layers, while the nano-layers of interest are depicted in dark teal, orange, and turquoise).

As highlighted by [52], the preparation of a material assembly cross-section is critically important for accurately visualizing nano features while minimizing the introduction of artifacts. In the first part of this review, the main preparation techniques are summarized, with particular attention given to the targeted sample surface or volume, the advantages and drawbacks of each method, and the related characterization tools (Figure 1, Table 3). It is important to first consider the characteristics of the structural features within the nano-layer in order to determine whether 2D or 3D analysis is more appropriate (Figure 1a). There are primarily five methods for preparing a material assembly containing nano-AdLs or nano-RLs: fracturing or cutting, mechanical polishing, ultramicrotome (UM) planarization or ultrathin sectioning, ion beam (IB) milling and polishing, and focused ion beam (FIB) milling and polishing (Figure 1b).

Sample fracturing or cutting involves breaking the sample with a sharp blade (or similar tool), resulting in a cross-section along the path of least mechanical resistance [53,54,55]. To better control the fracture path, a pre-notch can be applied to the sample before fracturing, which is then induced by applying a bending force [54]. It is noteworthy that this method yields the best surface quality in terms of cleanliness and sharpness when performed on a frozen sample prior to fracturing. This approach is fast and straightforward, allowing visualization of the coarse structure of layers within a material assembly [52,54,56,57]. However, it also introduces significant artifacts—including local material deformation, damage, and debris—leading to increased roughness [58]. Consequently, the detection of chemical contrast is non-optimal, making it difficult to distinguish different types of layers accurately within the assembly [52].

Mechanical polishing is another preparation method based on smoothing the material surface against abrasives (such as sandpaper, lapping films, or abrasive particle suspensions/pastes) under mechanical forces and lubrication [59]. This technique is routinely used to visualize layers in material assemblies [60,61,62], offering fewer artifacts compared to fracturing or cutting. However, inherent artifacts associated with mechanical polishing—such as local phase transformations, dislocations, stacking faults, deformations, streaking of polished layers, and filling of existing defects—are significant drawbacks of this method [52,59,63]. An important aspect of mechanical polishing tools is that they can also serve as preliminary sample thinning techniques for ion beam (IB) and focused ion beam (FIB) methods, enabling a substantial reduction in the time required to prepare thin or ultrathin sections [64,65].

The ultramicrotome (UM) technique is employed for surface planarization, reducing surface roughness, and sectioning samples. It involves the precise slicing of a small material piece using specialized knives—glass knives for trimming and thick sectioning, and diamond knives for fine sectioning—aiming to produce a very flat, planar surface and thin or ultrathin sections that can be retrieved for analysis [66,67,68]. Compared to the two previous methods, mechanical polishing and fracturing, UM significantly minimizes preparation-induced artifacts, which facilitates the analysis of chemical contrast. However, the volume of the targeted material surface is much smaller with this approach. Through the preparation of ultrathin sections, it becomes possible to analyze the internal structure of sample assemblies under transmitted radiation, which is essential for high-resolution techniques like TEM [69]. Nano-layers within material assemblies of various natures (organic or inorganic) have been successfully imaged from thin sections prepared by the UM method [70,71,72]. Additionally, this method can produce sections with submicron thickness suitable for techniques that require extremely flat surfaces, such as nanoSIMS [73].

Another method of interest for preparing the cross-section of a material assembly is ion beam (IB) milling and polishing, which aims to reduce surface roughness locally. This technique employs low-ion energy to finely polish the sample by removing loosely bonded surface atoms using a high-energy ion beam (typically Ar^+^ ions), thereby revealing the intrinsic atomic structure within the eroded region [74,75]. Additionally, this method is widely used for final sample thinning through low-angle ion milling, which allows the production of ultrathin sections following an initial pre-thinning step [74]. The pre-thinning procedure may involve mechanical polishing, magnetorheological polishing, or dimpling. For material assemblies containing multiple layers, IB milling has been successfully employed to prepare cross-sections suitable for microscopy observations [76,77].

When precise positioning below the micron scale and/or specific orientation for milling is required within a sample, the focused ion beam (FIB) technique integrated into a scanning electron microscope (SEM) chamber is the preferred method. SEM allows real-time control and monitoring of the FIB preparation process. FIB relies on ionizing a liquid metal, typically gallium (Ga), from a needle tip using an electric field. The resulting Ga^+^ ions are accelerated and focused on the sample surface via electrostatic lenses. When these ions collide with sample atoms, they cause sputtering of neutral and ionized atoms if their kinetic energy exceeds the surface binding energy, effectively milling the material [78,79,80]. However, this process can introduce surface artifacts such as Ga implantation, amorphization, lattice strains, and crystal defects. To minimize these artifacts, FIB milling can be performed at lower ion energies and incident angles, serving as a polishing step, although some artifacts may still persist. Further reductions in artifacts can be achieved through additional ion beam polishing procedures [81]. FIB has been successfully used to prepare cross-sections of material assemblies for microscopy imaging [69,77,82]. Additionally, FIB is commonly employed to fabricate needle-shaped samples required for atom probe tomography (APT) measurements [78]. It can also be used to extract thin sections from polished samples, with thicknesses ranging from submicron to a few micrometers. These thin sections are suitable for characterization techniques like nanoSIMS [83].

As discussed in this section, multiple methods can be employed—and sometimes combined—to prepare a cross-section of a material assembly for structural characterization. The choice of methods depends on the specific structural information required, the sample geometry, size, the location of the region of interest, and the potential artifacts introduced during preparation. Consulting with a sample preparation specialist is generally recommended to select the most appropriate approach, with the goal of minimizing the number of preparation steps and overall procedure time [77,84].

## 3. Techniques Providing 2D Structural Information

### 3.1. SEM

The most common techniques for acquiring 2D structural information are microscopy methods, with scanning electron microscopy (SEM) being a particularly powerful and versatile tool for analyzing the surface of materials. SEM provides detailed topographical and chemical composition insights. A key feature of SEM is its large depth of field—typically ranging from a few microns to several millimeters—which allows a significant portion of the sample surface to be in focus simultaneously. This capability facilitates three-dimensional imaging, greatly enhancing the understanding of a material’s structural properties. In SEM, an electron beam is focused onto the sample and interacts within a region known as the interaction volume, which generally spans a few microns (see [85] for a schematic). In this volume, primary electrons exchange energy with atoms in the material, producing various signals. The three main signals used for SEM analysis are as follows:

(i) Secondary electrons (SE): Emitted from the top part of the interaction volume, primarily used for high-resolution topography imaging;

(ii) Backscattered electrons (BSEs): Emitted from the upper half of the interaction volume, used for Z-contrast imaging;

(iii) X-rays: Emitted from the entire interaction volume, analyzed via energy-dispersive X-ray spectroscopy (EDS or EDX) for elemental composition [86,87].

Depending on factors such as the sample’s stability under electron irradiation, the size of the interaction volume, and SEM instrumental parameters, topographical imaging can achieve a spatial resolution in the order of 1 nm. When analyzing the cross-section of nano-AdLs or nano-RLs, such features can be precisely visualized using the SE imaging mode [52]. It is important to note that three types of SE signals are generated:

(i) SE1: From the region where the beam directly strikes the sample, offering the highest resolution;

(ii) SE2: Inelastically scattered BSEs from beneath the surface, with lower resolution than SE1;

(iii) SE3: Produced when BSEs strike the SEM’s objective lens pole piece or chamber walls, which can introduce noise.

**Figure 2:**
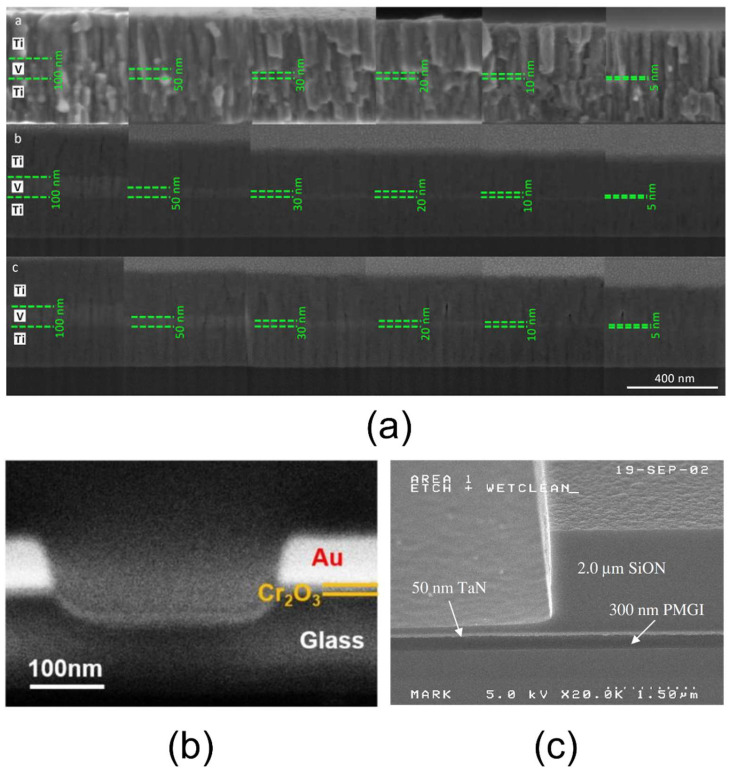
Examples of (**a**) nano-layer, (**b**) nano-AdL, and (**c**) nano-RL observed by SEM. (**a**) Ti/V/Ti multilayers as model materials with a V nano-layer of thickness varying from 5 nm to 100 nm [52]. (**b**) Cr_2_O_3_ adhesion layer between a Au film and a glass substrate for plasmonic applications (reproduced with permission [89], Copyright 2020, RCS). (**c**) Polymethylglutarimide (PMGI) as a release layer for microelectromechanical system (MEMS) resonators (reproduced with permission [90], Copyright 2005, IOP).

To optimize topographical resolution, SE3 signals should be minimized—either by using shielding made from low-SE-emission materials or by increasing the working distance. Additionally, operating at lower acceleration voltages reduces the interaction volume, improving SE emission from the surface [85,88]. SEM imaging of well-prepared cross-sections, obtained via ion beam (IB) or focused ion beam (FIB) methods, enables layer thickness measurements ranging from a few nanometers to several hundred nanometers (see Figure 2a–c). This approach also allows an assessment of layer homogeneity—such as the presence of inclusions or porosity—and the qualitative evaluation of bonding quality at the interfaces. The presence of interfacial porosity, for example, suggests a non-optimal adhesion. However, any debonding artifacts introduced during the preparation must be excluded from the analysis. Chemical composition imaging using BSEs and EDS generally offers lower resolution than topographical SE imaging because these signals originate from larger volumes—around 1 µm—which is insufficient for detailed nano-layer characterization within a material assembly. Nonetheless, BSEs and EDS are valuable for qualitative elemental analysis; for instance, EDS can verify the presence or absence of specific elements in a nano-layer when detailed chemical mapping is not required. It has been reported that BSE resolution can be marginally improved by applying a specimen bias, which increases BSE velocity and collection efficiency [86]. Finally, a limitation of SEM involves imaging organic nano-layers; such materials can be damaged or degraded under electron beam exposure, making high-magnification SE imaging infeasible for organic layers. To mitigate this issue, SEM imaging can be performed using a low acceleration voltage, low beam current, and a reduced spot size. Additionally, it is advisable to adjust the focus, astigmatism, and brightness/contrast in a non-critical area before moving to the region of interest for rapid image acquisition. Applying a conductive coating can also help reduce beam-induced degradation.

### 3.2. TEM

To achieve high-resolution 2D imaging and a detailed chemical analysis of nano-AdLs or nano-RLs, transmission electron microscopy (TEM) is the preferred technique. Additionally, TEM enables the acquisition of electron diffraction patterns, providing valuable information about the crystalline structure of specific regions. In TEM, a high-voltage electron beam is generated and focused onto an ultrathin sample section positioned on a grid using condenser lenses. As electrons pass through the sample, imaging lenses project the transmitted electrons onto a fluorescent screen or camera system, producing either magnified images or diffraction patterns [91]. The transmitted electrons include both scattered electrons—deflected by interactions with sample atoms—and unscattered electrons. Several types of images can be obtained:

(i) Bright-field (BF) images: These are formed by detecting mainly unscattered electrons. In BF images, regions appearing dark correspond to high-atomic-number (high-Z) areas that strongly scatter electrons, while bright regions correspond to low-Z regions with less scattering. Sample thickness variations also influence image contrast; thicker areas scatter more electrons and appear darker. The presence of crystalline regions causes enhanced diffraction in specific directions, affecting contrast depending on crystal orientation.

(ii) Dark-field (DF) images: These are created by collecting only the scattered electrons. Here, areas that scatter electrons strongly appear bright, while regions that scatter minimally look dark. DF imaging provides high sensitivity and low noise, making it especially useful for investigating crystallographic features such as lattice structures, dislocations, stacking faults, and interfaces between crystalline domains [92,93,94].

**Figure 3:**
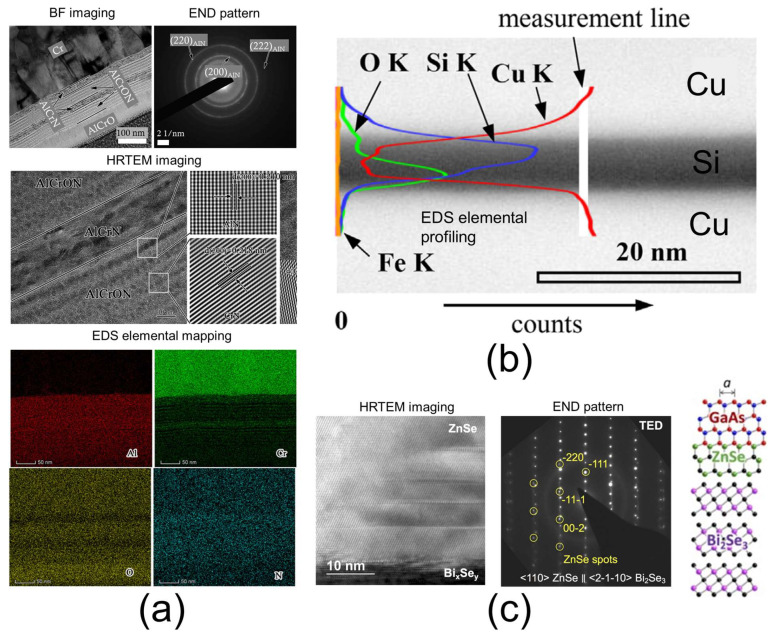
Examples of (**a**) nano-layer, (**b**) nano-AdL, and (**c**) nano-RL observed by TEM. (**a**) Multi-layer coating for concentrating solar power (CSP) applications with a Cr layer used as an IR reflector, alternative AlCrN/AlCrON layers as absorbers, and an AlCrO layer as an anti-reflective layer, imaged by BF, HRTEM, and EDS and characterized by END. BF imaging enables the characterization of the thickness of different layers ranging from 22 nm to 270 nm. END patterns prove that the different layers comprise an amorphous phase (intense central diffusion) encapsulating nanocrystals (diffraction rings). HRTEM enables the identification of the presence of nanocrystals of CrN and AlN in AlCrN and AlCrON layers and nanocrystals of Al_2_O_3_ and Cr_2_O_3_ in AlCrO. EDS imaging enables the visualization of the elemental distribution within the different layers of the material assembly [97]. (**b**) Nano-AdL of amorphous Si between two Cu electrodes. EDS elemental profiling enables the characterization of the elemental composition of the assembly, with attention focused on O. This element is highly detected at one Cu-Si interface due to the oxidation of Si [101]. (**c**) Development of a nano-RL made of Bi_2_Se_3_ and ZnSe, on which a layer of GaAs is deposited for semiconductor applications. The GaAs layer can be mechanically exfoliated from the layered Bi_2_Se_3_ substrate. This mechanism is possible thanks to the presence of ZnSe, which enables a lattice-matched template for the growth of GaAs (111), and by the fact that ZnSe is epitaxially aligned to the layered Bi_2_Se_3_ substrate, as shown by HRTEM imaging and END (reproduced with permission [102], Copyright 2020, Elsevier).

An important aspect to note is that the contrast in dark-field (DF) images strongly depends on the crystal’s spatial orientation, as the electron scattering intensity varies with direction relative to the crystal lattice. This orientation-sensitive contrast enhances the ability to analyze crystallographic features. A third important TEM technique is electron nanodiffraction (END), which involves recording the scattered electrons to produce diffraction patterns. These patterns appear as spots for monocrystalline regions or rings for polycrystalline materials [91]. The diffraction spots or rings result from Bragg reflections of the crystalline planes within the observed region. Analyzing the position of these features relative to the transmitted beam allows for the determination of lattice parameters and crystal orientation. Finally, high-resolution TEM (HRTEM) provides atomic-scale imaging by recording both unscattered and scattered electron waves. This interference-based imaging reveals the atomic structure of the sample, enabling detailed analysis of lattice arrangements and defects [95]. HRTEM is also known as phase-contrast imaging. However, interpreting HRTEM images can be complex because various factors—such as local sample thickness variations, crystal orientation, and spherical aberrations of the TEM lens—can influence the phase contrast. The typical spatial resolution of TEM imaging is around 0.1 nm. Another important imaging modality is energy-dispersive X-ray spectroscopy (EDS), which provides elemental mapping of the material assembly [96,97]. EDS works by collecting X-rays emitted from the region illuminated by the focused electron beam, which can be focused to a diameter of just a few nanometers. The characteristic X-ray energy corresponds to specific elements, enabling their identification. Generally, elements with atomic numbers from 4 (Be) to 92 (U) can be detected with a detection limit around 0.1 wt% [98]. Additionally, electron energy loss spectroscopy (EELS) is another elemental analysis technique available in TEM. Typically, EELS is more sensitive to low-Z elements, whereas EDS is more sensitive to high-Z elements. A notable advantage of EELS is its ability to provide information regarding the bonding state of elements, which cannot be obtained through EDS [99,100]. The spatial resolution of TEM-EDS or TEM-EELS usually ranges between 1 nm and 20 nm. Examples of TEM imaging for multilayer materials and assemblies containing nano-AdLs or nano-RLs are shown in Figure 3a–c. Similar to SEM, TEM can determine the thickness of the layers of interest via bright-field (BF) imaging (Figure 3a,b) [97,101]. Moreover, HRTEM and diffraction techniques enable the visualization and identification of the crystalline phases within the layers—also allowing the detection of amorphous regions (Figure 3b,c) [101,102]. These analyses are particularly useful for verifying whether the crystalline parameters of a layer match those of neighboring layers or contact regions (Figure 3c) [102]. Finally, EDS analysis allows verification of the elemental composition within the layer of interest. Due to the high spatial resolution of TEM-EDS, elemental profiling and mapping can be performed to identify phenomena such as oxidation at interfaces (Figure 3b) [101] and diffusion mechanisms between layers. However, TEM imaging has several limitations to consider. First, as discussed in Section 2 (Table 3), sample preparation is complex, requiring a high level of expertise, and can introduce artifacts that may lead to misinterpretation of the structure. Second, the analyzed cross-section represents only a small volume, which may not be fully representative of the entire sample; multiple sections should be examined to obtain more robust data. Third, TEM provides 2D projections of a 3D structure—the ultrathin section—meaning that observed features may be superimposed from different phases along the section’s thickness, potentially complicating the interpretation of layer interfaces and internal structures. Fourth, the high electron energy can cause sample damage, especially in organic materials, making the high-resolution imaging of organic layers particularly challenging. To address this issue, three methods have been successfully developed: (i) low-dose TEM using direct-detection electron-counting (DDEC) cameras instead of scintillator-based charge-coupled device (CCD) cameras [103]; (ii) low-voltage TEM, operating at accelerating voltages below 30 kV [104]; and (iii) cryo-TEM, in which samples are cooled to cryogenic temperatures [105].

### 3.3. AFM

**Figure 4:**
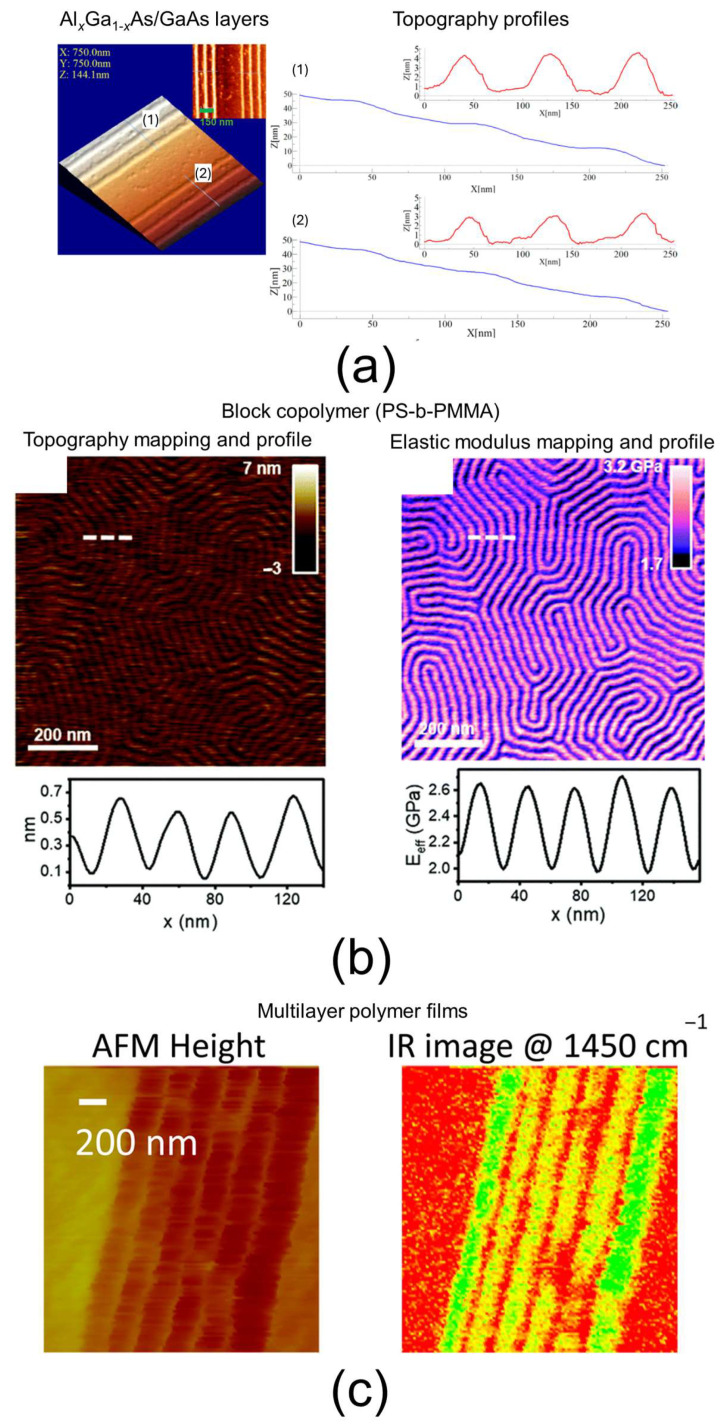
Examples of nano-layers or nano-lamellae characterized by AFM. (**a**) Al*_x_*Ga_1−*x*_As/GaAs superlattice layers for semiconductor applications investigated in topography with conventional AFM (in blue: non-corrected profiles; in red: corrected profiles—two cases (1 and 2) are analyzed) (reproduced with permission [112], Copyright 2013, ACS). (**b**) PS-b-PMMA block copolymer as a model ordered lamellar material studied with AFM equipped with a nanomechanical mode [113]. (**c**) Multilayer polymer film analyzed by AFM-IR (reproduced with permission [114], Copyright 2017, ACS).

In atomic force microscopy (AFM), a sharp tip attached to the end of a cantilever is scanned across the surface of interest. The deflection of the cantilever is directly related to the local, nanoscale properties of the surface. By performing line-by-line scans, AFM produces a 2D map of various surface characteristics, including electrical, magnetic, mechanical, and topographical features [106,107]. AFM was invented in 1986 and is part of the broader family of scanning probe microscopy (SPM) techniques [108]. One major advantage of AFM is its versatility in evaluating a wide range of surface properties. Different modes and sub-modes have been developed to target specific local surface characteristics. For example, conductive AFM (c-AFM) is a sub-mode within contact AFM where a direct current bias is applied between a conductive tip and the sample surface, enabling the measurement of local electrical conductivity. For mechanical property investigations, numerous nanomechanical modes have been introduced, broadly categorized into quasi-static and dynamic modes [107]. Recently, a powerful and innovative technique called AFM–infrared spectroscopy (AFM-IR) has been developed, combining the chemical analysis capabilities of infrared spectroscopy with AFM’s nanoscale resolution [109]. Another key advantage of AFM is its ability to operate in diverse environments—including vacuum, air, and liquids—and across a broad temperature range, from cryogenic to high temperatures. This versatility is especially advantageous for studying biological structures or molecules in aqueous media, which is often challenging with other microscopy techniques. Finally, the lateral resolution of AFM depends primarily on the contact area between the tip and the sample, which is influenced by the tip apex radius and surface properties such as stability, adhesion, and the presence of protrusions. These factors can affect resolution quality and may introduce artifacts [110]. Considering that the most advanced AFM tips have an apex radius of about 1 nm, a lateral resolution of approximately 1 nm is generally regarded as the fundamental limit for AFM measurements [110]. Typical lateral resolutions achieved with conventional tips—especially in standard AFM, conductive AFM (c-AFM), and nanomechanical modes—range from about 5 nm to 15 nm. For AFM-IR, the lateral resolution is usually in the range of 20 nm to 100 nm [111]. In the case of nano-RLs or nano-AdLs, AFM can provide detailed morphological images, offering qualitative insights into layer thickness, homogeneity, and bonding quality. When equipped with conductive or nanomechanical modes, AFM can further analyze the layers’ electrical conductivity and viscoelastic properties, respectively. Moreover, the topographical, electrical, and viscoelastic profiles of the interfacial regions between the nano-layer and the surrounding phases can be obtained, providing additional structural information about the material. Due to its lower lateral resolution, AFM-IR is primarily used to obtain qualitative information—such as identifying materials via their IR spectra or confirming the presence or absence of specific chemical bonds—from nano-RLs or nano-AdLs. Some examples of AFM analyses of nano-layers are shown in Figure 4. In the first example (Figure 4a), conventional AFM was used to measure the thickness of Al*_x_*Ga_1−*x*_As/GaAs superlattice layers for semiconductor applications. Topographical profiles were extracted and corrected, revealing average thicknesses of approximately 8.5 nm in one case and 6.5 nm in another [112]. In the second example (Figure 4b), AFM equipped with a nanomechanical mode was employed to investigate a poly(methyl methacrylate)-block-polystyrene (PS-b-PMMA) copolymer as a model ordered lamellar material. Elastic modulus mappings and profiles demonstrated that the two phases exhibit distinctly different elastic properties, providing additional structural insights [113]. In the final example (Figure 4c), AFM-IR was employed to study a multilayer polymer film, focusing on the mapping of a specific IR band at 1450 cm^−1^ [114]. This characterization revealed that the multilayer consists of alternating layers: 200 nm thick and 20 nm thick. Despite its versatility and high sensitivity, AFM does have certain limitations. First, the tip–sample interactions must be well understood and carefully controlled by the operator to avoid misinterpretation of the results. This is particularly critical in tapping mode, where distinguishing between attractive and repulsive regimes is essential for accurate morphology imaging [106]. Second, the sharp nanoscale tip used as the measurement probe is a consumable; its shape and dimensions can change between experiments, which can influence the in-plane resolution. Moreover, especially in contact or indentation modes, the tip may suffer damage (wear, local spallation), potentially altering the results during a mapping session. Additionally, the tip can become contaminated by nano-objects or nano-layers present on the sample surface, forming a third body on the tip and further affecting measurement accuracy. Consequently, proper surface preparation and maintaining surface cleanliness are critical for obtaining reliable AFM data. Finally, accurately determining the tip shape—either directly or indirectly—is necessary for the precise evaluation of nanomechanical properties [107]. A few calibration steps, such as accurately determining the cantilever’s spring constant, are essential to ensure reliable AFM measurements. Since the evaluation of mechanical properties often relies on models based on the Hertz contact theory, it is important to adhere to its general constraints—for example, analyzing flat surfaces and accurately estimating the sample’s Poisson’s ratio. Additionally, selecting the appropriate mechanical model—such as the Derjaguin–Muller–Toporov (DMT) model or others—must be tailored to the specific experimental conditions. Finally, a limitation of AFM is its relatively low acquisition rate compared to techniques like SEM. However, recent developments in high-speed AFM offer promising avenues for faster and more efficient measurements, expanding its applicability to dynamic or time-sensitive studies [115].

### 3.4. HIM

**Figure 5:**
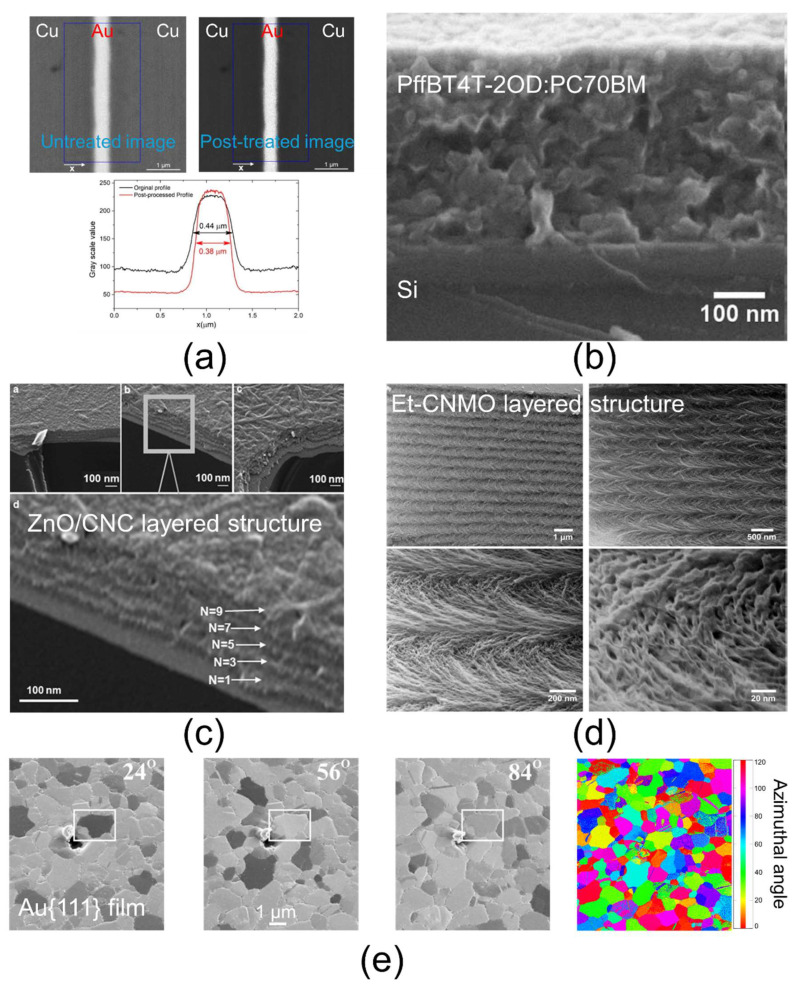
Examples of material samples imaged by HIM using the SE detection mode. (**a**) Imaging of a model sample consisting of a Au nano-layer sandwiched between two Cu layers and estimation of Au nano-layer thickness from an untreated and post-treated image [122]. (**b**) Imaging of a poly[(5,6-difluoro-2,1,3-benzothiadiazol-4,7-diyl)-alt-(3,3′′′-di(2-octyldodecyl)-2,2′;5′,2″;5″,2′′′-quaterthiophen-5,5′′′-diyl)]:phenyl C71 butyric acid methyl ester (PffBT4T-2OD:PC70BM) nano-film deposited on a silicon substrate for organic photovoltaic applications [123]. (**c**) Imaging of ZnO/CNC layered structure aimed at harvesting thermoelectric energy [124]. (**d**) Imaging of layered structure of nematic mesoporous organosilicas with ethylene groups (Et-CNMO), targeting catalysis, sensors, and fuel-cell applications (reproduced with permission [125], Copyright 2013, RSC). (**e**) Imaging of a model polycrystalline Au{111} film recorded at different sample rotation angles around the surface normal [111] (angles of 24°, 56°, and 84°), highlighting the channeling effect and corresponding orientation mapping of the grains around the [111] surface normal (the color of each grain corresponds to the azimuthal angle for which a <110> direction in this grain is parallel to the helium ion beam) [120].

Scanning helium ion microscopy (HIM) is a powerful characterization technique operating at the nanoscale and sub-nanoscale for high-resolution imaging, patterning, and chemical analysis. Details about the HIM method are available in various reviews [116,117,118]. Briefly, HIM employs a biased, cooled sharp needle that induces the ionization of a helium (He) or neon (Ne) gas injected near the tip. The resulting He^+^ or Ne^+^ primary ions are accelerated toward the sample, with probe sizes of less than 0.5 nm for He+ and approximately 1.5 nm for Ne^+^. When these ions strike the sample, they produce secondary electrons (SE), and a fraction of the incident ions are backscattered as helium atoms and ions (BSHe). By collecting these signals with dedicated detectors, HIM generates different imaging contrasts, achieving a spatial resolution of about 0.5 nm for SE imaging (slightly lower in BSHe mode). Certain materials, such as direct band-gap semiconductors and rare-earth-doped garnets, can emit photons under ion irradiation, enabling ionoluminescence imaging and analysis [119]. Since the interaction volume in HIM is smaller than in SEM—especially near the surface—SE imaging in HIM permits higher spatial resolution compared to SEM SE imaging. HIM is also capable of investigating insulating samples without surface charging artifacts, thanks to the use of a charge-neutralizing electron flood gun. Moreover, because the ion beam induces considerably less surface damage than an electron beam, HIM is particularly suitable for low-Z (low-atomic-number) materials. The primary use of SE detection in HIM is to produce topographical images with enhanced resolution. One notable advantage over SEM is HIM’s five times higher depth of field, allowing for more extensive surface detail in three dimensions. Additionally, crystallographic texture information can be obtained quantitatively by analyzing the so-called channeling pattern—a geometric representation of the crystal lattice symmetry from the perspective of the incident ion beam. Varying the beam’s incident direction causes changes in the channeling pattern, enabling the determination of individual grain orientations through orientation mapping techniques [120]. Images with chemical contrast in HIM are obtained through the detection of BSHe signals. It is important to note that while BSHe mode also enables crystallographic texture characterization, the signal-to-noise ratio in this mode is generally lower compared to the SE mode. Several examples of nano-layer imaging by HIM are presented in Figure 5: inorganic layers are shown in Figure 5a,c,d, and organic layers are shown in Figure 5b. All these images were acquired using the SE mode. A key structural insight provided by HIM is the estimation of nano-layer thickness (Figure 5a), as well as the assessment of layer homogeneity and bonding quality—such as the presence or absence of interfacial porosity—since HIM allows the scanning of large sample areas with high resolution. As demonstrated in Figure 5d, HIM can resolve nanoscale structural details that are challenging to achieve with SEM. Regarding crystallographic texture analysis, an example of the channeling effect is shown in Figure 5e for a metallic film, where grain orientations were determined from SE imaging. Although chemical contrast imaging is achieved via BSHe detection, elemental identification by EDS is not possible with the He ion beam, as it does not produce X-ray emission. To overcome this limitation, ongoing developments aim to integrate analytical capabilities into HIM systems by detecting and analyzing sputtered secondary ions with mass spectrometry. Notably, methods such as HIM secondary ion mass spectrometry (HIM-SIMS) [118] and HIM time-of-flight secondary ion mass spectrometry (HIM-ToF SIMS) [121] are emerging technologies. Two significant limitations of HIM are its complexity and high cost, which restrict access primarily to well-resourced laboratories. Consequently, widespread availability remains limited.

### 3.5. AES

**Figure 6:**
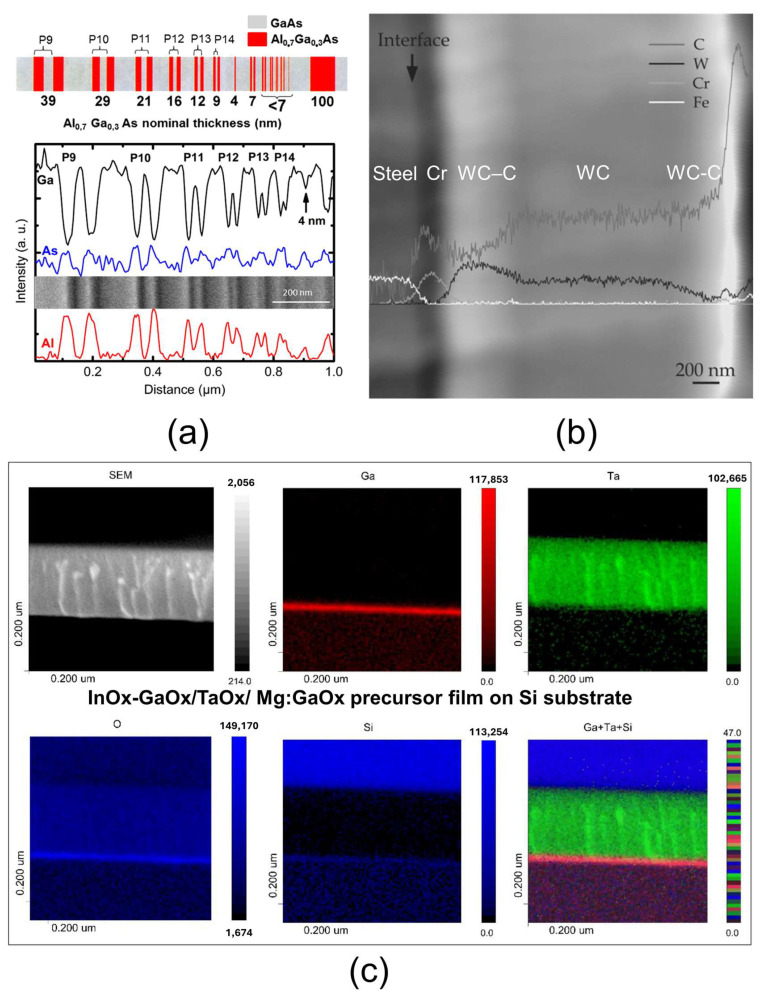
Examples of nano-layers analyzed by AES. (**a**) Elemental profiling of semiconducting Al_0.7_Ga_0.3_As/GaAs multilayers based on Auger intensity of Al KLL, As LMM, and Ga LMM peaks (insert: SEM image) (reproduced with permission [127], Copyright 2013, Elsevier). (**b**) Elemental profiling along a multilayer coating made of WC-C deposited on steel (Fe-Cr) (reproduced with permission [129], Copyright 2006, Springer). (**c**) Elemental mapping and corresponding SEM image of a InO_x_-GaO_x_/TaO_x_/Mg:GaO_x_ precursor film on a Si substrate for semiconductor-based solar energy conversion devices [130].

Auger electron spectroscopy (AES) provides detailed elemental composition with a spatial resolution of approximately 10 nm and a depth of analysis ranging from about 1.5 nm to 15 nm, making it particularly well suited for nanomaterial characterization. The technique’s detection limit is around 0.1 wt% to 1 wt%, depending on the element, but elements with fewer than three electrons—such as hydrogen and helium—are not detectable. AES is based on detecting Auger electrons emitted by sample atoms when excited by a high-energy electron beam. The kinetic energy of these electrons is characteristic of the element present at the surface, enabling element identification through spectroscopic analysis. The high resolution of AES arises from the fact that Auger electrons escape from the extreme surface layer (1.5–15 nm) of the sample. A focused electron beam with a diameter of approximately 10 nm at the surface is used. This spectroscopy is implemented in a scanning Auger microscope (SAM) operating under vacuum conditions, which generally limits measurements to conductive samples. However, charge neutralization devices can now be used to analyze insulating materials. In addition to spectral analysis, SAM can also produce surface imaging based on secondary electron detection. An important feature of SAM is its capability for in-depth elemental analysis through controlled ion sputtering, allowing for in-depth profiling of multilayer structures [126,127,128]. AES has been extensively employed to analyze material assemblies with nanolayers exhibiting various functionalities. In Figure 6a, the potential of AES for nanoscale chemical profiling is demonstrated using a model Al_0.7_Ga_0.3_As/GaAs multilayer sample, featuring Al_0.7_Ga_0.3_As lines with thicknesses ranging from 4 nm to 39 nm deposited on a GaAs substrate. The profiling of Ga, As, and Al across these lines is successful even for very thin features, highlighting the high spatial resolution of AES [127]. Another example shown in Figure 6b involves a multilayer coating composed of WC-C deposited on steel (Fe-Cr). The elemental profiling of C, W, Cr, and Fe enables the identification of thin coating layers, such as a Cr layer at the steel surface, as well as interfacial regions where the elemental composition exhibits a gradient, for instance between steel and the Cr layer [129]. A third example (Figure 6c) illustrates how AES is used for the elemental mapping of an InOx-GaOx/TaOx/Mg:GaOx precursor film deposited on a Si substrate. This analysis visualizes a nanometer-thick GaOx layer atop a micrometer-scale TaOx layer [130]. However, AES does have certain limitations. First, samples that are unstable under electron beam irradiation cannot be analyzed. Second, since AES provides elemental information only from the extreme surface, surface contamination can affect the results. Lastly, analyzing insulating materials is challenging due to charging effects, and thus the use of charge compensation techniques is required.

### 3.6. nanoSIMS

**Figure 7:**
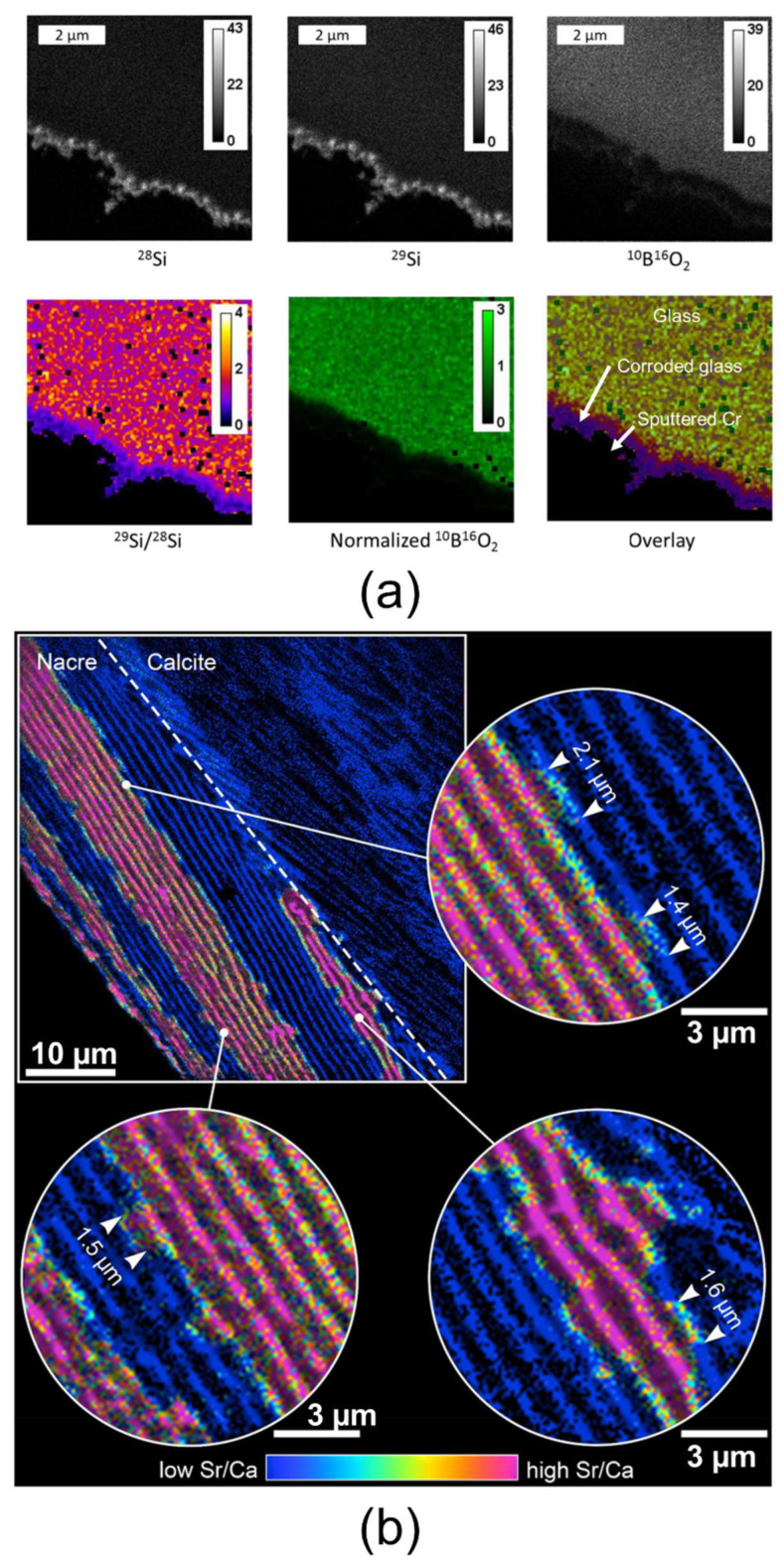
Examples of nanoSIMS analyses conducted in cases of materials having internal layers. (**a**) Ion maps of ^28^Si^−^, ^29^Si^−^, ^10^B^16^O_2_^−^, ^29^Si^−^/^28^Si^−^, and ^10^B^16^O_2_^−^/(^29^Si^−^/^28^Si^−^), and overlaid image in the case of SON68 glass with a corrosion layer used for nuclear waste immobilization (reproduced with permission [135], Copyright 2014, John Wiley and Sons). (**b**) Maps showing ^88^Sr/^40^Ca isotope abundance in the case of the nacre biomineral [136].

Nanoscale secondary ion mass spectrometry (nanoSIMS) allows for the elemental and isotopic analysis of material assembly cross-sections when the region of interest spans several hundred nanometers. The current state-of-the-art nanoSIMS instruments achieve a lateral resolution of approximately 30 nm, with depth resolutions ranging from sub-nanometer to tens of nanometers. This technique offers extremely high sensitivity, with detection limits typically between parts per million (ppm) and 1 atomic percent (at%) depending on the targeted element. For optimal operation, the sample must withstand high vacuum conditions and possess a very flat surface. High electrical conductivity is also essential to ensure accurate data acquisition, which can often be achieved by smoothing the surface and depositing a metallic coating if necessary. Regarding sample dimensions, the technique can analyze sections from ultrathin slices to several millimeters thick, with lateral dimensions varying from a few microns (requiring a suitably large substrate mounted on the sample holder) up to several millimeters. NanoSIMS functions by sputtering the sample surface with a primary ion beam, analyzing the emitted ionized particles via a mass spectrometer. This results in detailed elemental and isotopic maps—from hydrogen to uranium—across the analyzed area. It is important to note that nanoSIMS operates in a dynamic sputtering mode: a significant volume of material is removed, producing highly fragmented secondary ions with limited molecular information, making the analysis inherently destructive. An advantage of nanoSIMS is its ability to conduct in-depth chemical profiling, enabling three-dimensional chemical mapping of the sample. Two types of primary ions are commonly used: O^−^ (or O^2−^) for detecting positive secondary ions (such as Na, K, Al, Mg, Ca, Mn, Fe, Ni, Co, Cu, Zn, and Mo), and Cs^+^ for negative secondary ions (such as H, C, O, N, P, S, and Si) [131,132,133,134]. NanoSIMS applications are shown in Figure 7a,b. The technique in Figure 7a was used to identify the size and composition of a ~500 nm corrosion layer on the surface of specialized glass used in nuclear applications by tracking different elemental distributions and ratios [135]. In Figure 7b, by analyzing isotopic ratios (e.g., ^88^Sr/^40^Ca), nanoSIMS provided insights into the growth mechanisms of individual nacre layers in biominerals [136].

### 3.7. TERS

**Figure 8:**
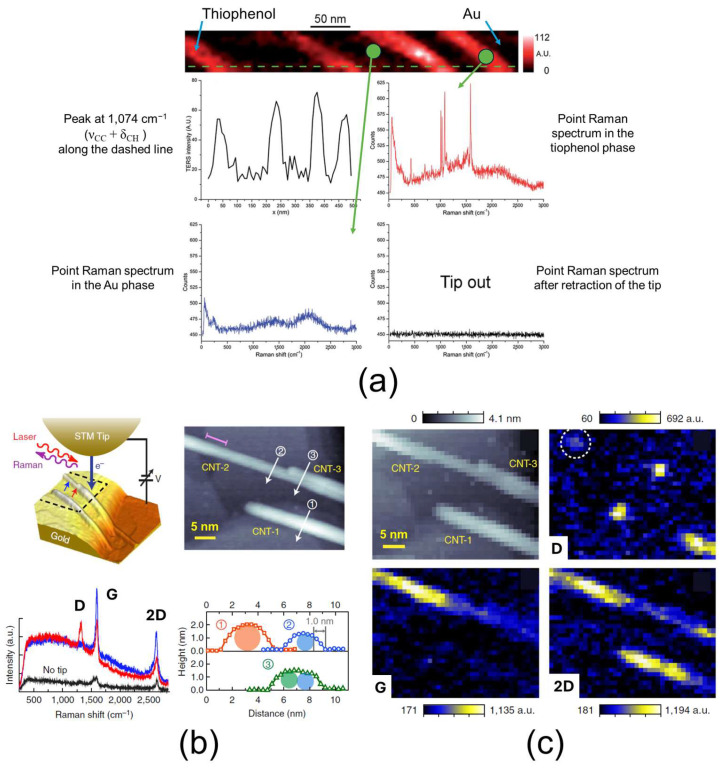
Examples of TERS analyses conducted in the case of (**a**) a model material consisting of thiophenol nanopatterns and (**b**,**c**) carbon nanotubes. Both materials are deposited on a gold substrate. (**a**) shows the STM-TERS chemical mapping of the thiophenol nanopatterns based on the intensity of the Raman peak recorded at 1074 cm^−1^ (ν_CC_ + δ_CH_), the profiling of this peak crossing different thiophenol nanopatterns, and point Raman spectra in the thiophenol and gold regions [139]. (**b**) represents a schematic illustration of the TERS method; an STM image of three carbon nanotubes, with three lines (with numbers 1 to 3) where topography measurements were conducted; Raman spectra recorded on one carbon nanotube (referred as CNT-2), showing the typical three Raman peaks of sp^2^ carbon materials (referred as D, G, and 2D) and topography profiles along the three lines [142]. (**c**) shows the simultaneous STM topography images recorded during TERS mappings, and TERS mapping of the integrated area of the Raman peaks D, G, and 2D [142].

Tip-enhanced Raman spectroscopy (TERS) is a powerful technique that enables both topographical and chemical mapping of a material at the nanoscale. It combines the principles of scanning probe microscopy (SPM) and Raman spectroscopy. Three types of SPM are commonly employed for TERS: atomic force microscopy (AFM), scanning tunneling microscopy (STM), and shear force or normal force microscopy. In TERS, a metal or metal-coated sharp tip (typically with a radius of 20–40 nm) is illuminated by a laser, which induces a localized surface plasmon resonance and the lightning rod effect. This creates a highly confined electromagnetic field at the tip apex—called a “hotspot”—which amplifies the Raman signals from molecules within this region by factors of 10^4^ to 10^6^. The Raman scattered light is collected via a microscope objective coupled with a spectrophotometer. State-of-the-art TERS systems can achieve a sub-nanometer spatial resolution for imaging and Raman analysis, enabling studies down to the level of single molecules. However, most typical measurements are performed with a lateral resolution in the range of 10–50 nm. The penetration depth of TERS is approximately 10 nm, indicating that it probes only the extreme surface layer of the sample. The resolution primarily depends on the size, shape, and material of the tip apex, which determines the hotspot size. One significant advantage of TERS is that it is a non-destructive technique compatible with ambient conditions. Nonetheless, it requires very flat samples since the technique relies on SPM contact. Typically, a nano-object (the sample) is transferred onto a gold substrate for analysis. For layered materials, ultrathin sections can be prepared via ultramicrotomy, deposited on clean glass coverslips, and analyzed via TERS. These sections must be optically transparent, exhibit low fluorescence background, and produce distinguishable Raman spectra [137]. It is important to note that TERS is effective only for Raman-active molecules or materials: they must undergo polarization changes during vibrational modes excited by the hotspot electromagnetic field, which is not possible for fcc or bcc metals. The polarization reflects the ease of electron distortion, influenced by the material’s chemical environment and structural modifications caused by stress or heat. A Raman spectrum acquired via TERS provides rich structural information. The position and relative intensity of bands help identify the material or molecule, assess crystalline phase composition, and detect the presence of amorphous versus crystalline regions. The spatial mapping of Raman bands can reveal structural homogeneity or heterogeneity across the sample. Additionally, shifts, broadening, or changes in band intensities can track structural rearrangements induced by mechanical stress or thermal treatment [138,139,140,141]. Two examples of TERS application are shown in Figure 8a–c. In Figure 8a, TERS is used to analyze thiophenol nanopatterns on gold, demonstrating nanoscale spatial resolution when recording characteristic spectra [139]. In Figure 8b,c, TERS mapping of carbon nanotubes on gold reveals heterogeneity in the Raman response at the nanometer scale [142]. In both cases, TERS enables the acquisition of the characteristic Raman spectrum of the material of interest with nanoscale spatial resolution, whether for point measurements, line profiles, or mapping. In the case of the nanotubes, TERS mapping revealed that the Raman response was heterogeneous at the nanoscale (Figure 8c).

### 3.8. SXRSM

**Figure 9:**
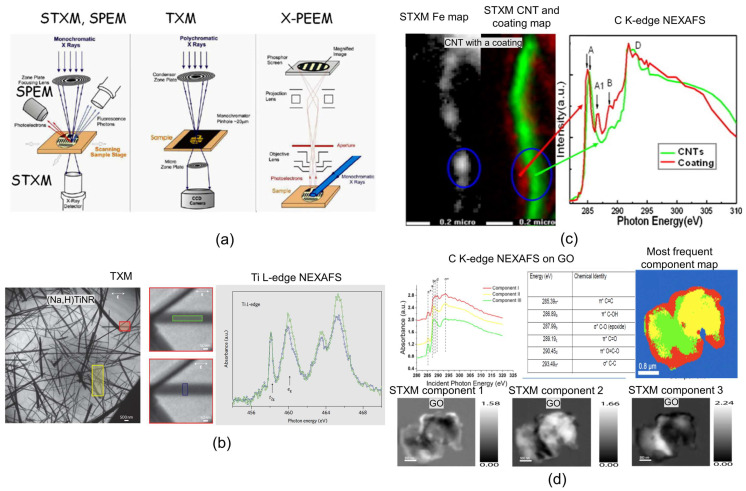
(**a**) Schematics of the different types of SXRSM (reproduced with permission [143], Copyright 2015, Elsevier) and examples of SXRSM analyses conducted in the case of (**b**) crystalline sodium titanate nanoribbons (Na,H)TiNR, (**c**) coated carbon nanotubes (CNT), and (**d**) graphene oxide (GO). (**b**) represents the TXM imaging of (Na,H)TiNR, with high-magnification images (rectangle red) representing the areas where NEXAFS analysis was performed, and the Ti L-edge NEXAFS spectrum of the 2 selected areas of (Na,H)TiNR in two spatial orientations shown by the rectangles green and blue (the two main first absorption peaks are assigned to the two sublevels of the Ti 3d band, split into t_2g_ and e_g_ symmetries) [155]. (**c**) shows the STXM map of Fe, a CNT, and a coating, as well as the C K-edge NEXAFS spectrum recorded in the CNT and coating areas (the main absorption peaks A, B, and D for the CNT are assigned to the π* excitation of C=C bonds, the transitions to sp^3^-hybridized states, and σ* excitation, respectively, while the main absorption peaks in the CNT coating are attributed to a shifting of A absorption, the appearance of a new absorption peak A1, and the enhancement of B absorption) (reproduced with permission [156], Copyright 2013, Elsevier). (**d**) represents the C K-edge NEXAFS spectrum of the three identified components of GO, the absorption peak assignment, a map representing the most frequent component in GO, and an STXM map of the three components (the image color legend is the optical density) (reproduced with permission [157], Copyright 2013, ACS).

As reported in ref. [143], soft X-ray spectromicroscopy (SXRSM) comprises a family of techniques that allow both imaging and spectroscopic analysis with a lateral resolution typically between 10 nm and 100 nm. These techniques utilize tunable soft X-ray energies (50 eV < hν < 3000 eV) generated from a synchrotron light source. Access to SXRSM methods generally requires a scientific proposal due to the reliance on specialized facilities, although some services may be available at synchrotrons where accessible. This family includes full-field transmission X-ray microscopy (TXM), scanning transmission X-ray microscopy (STXM), X-ray photoemission electron microscopy (X-PEEM), and scanning photoemission microscopy (SPEM), whose principles are illustrated in Figure 9a. The image contrast in these techniques depends on the specific method and the X-ray energy used. In TXM and STXM, imaging can be performed in absorption-contrast mode or phase-contrast mode, depending on the configuration of detection optics and X-ray energy [144,145]. In X-PEEM and SPEM, images result from the detection of secondary electrons, with contrast proportional to the X-ray absorption in the near-surface region. In these methods, the contrast arises from variations in electron emission that are influenced by the surface topography, including effects like emission enhancement and shadowing [146,147]. Both techniques typically map the spatial variation in the energy and intensity of electrons emitted from core or valence levels, which are sensitive to surface features. These four imaging approaches are often coupled with near-edge X-ray absorption fine structure (NEXAFS), also referred to as X-ray absorption near-edge structure (XANES), for chemical analysis in transmission mode. NEXAFS investigates how X-rays are absorbed by atoms near their absorption edge, revealing information about the electronic structure. When an X-ray photon excites an electron from a core level, it creates a core hole. The subsequent filling of this hole by a higher-energy electron results in the emission of either a fluorescent photon or an Auger electron—a process known as decay [148,149]. Analysis of the resulting absorption spectrum enables detailed insights into the local electronic environment of the atom, including oxidation state, coordination chemistry, bond distances, coordination number, and neighboring species. This comprehensive chemical information enhances our understanding of material composition and bonding at the nanoscale [150]. NEXAFS can be performed on both crystalline and non-crystalline materials, and for nearly all elements in the periodic table, except hydrogen. As detailed in Refs. [148,151], since NEXAFS employs polarized X-rays, it allows for the characterization of the orientation of specific electronic structures and associated chemical bonds by rotating the sample relative to the beam. This technique exhibits high sensitivity, and is capable of detecting and analyzing atoms at concentrations as low as sub-ppm levels. The lateral spatial resolution of NEXAFS is primarily determined by the size of the X-ray beam spot on the sample surface and the acceptance area of the detector; recent studies report resolutions around 30 nm [152]. For the optimal coupling of NEXAFS with imaging techniques such as STXM, TXM, X-PEEM, and SPEM, the sample usually needs to be ultrathin and mounted on a suitable holder—specifically, a conductive holder for non-conductive samples in SPEM and X-PEEM. Recently, the development of X-ray spectro-ptychography (XRSP) has significantly improved the spatial resolution of SXRSM. XRSP reconstructs images from multiple coherent diffraction patterns recorded over overlapping regions, achieving lateral resolutions as fine as 3 nm with enhanced detail clarity. Both absorption and phase-contrast images can be obtained through X-ray ptychography (XRP) using either absorption or phase-contrast mechanisms. Notably, XRSP enhances the quality of NEXAFS spectra and increases spatial resolution further—up to approximately 20 nm—making it especially powerful for analyzing small, dense phases [143,153,154]. Several examples of SXRSM analysis are illustrated in Figure 9b–d. One combines TXM with NEXAFS to study crystalline sodium titanate nanoribbons ((Na,H)TiNR). By tracking the absorption peak intensities of Ti, subtle spectral differences are observed along two spatial directions of interest (Figure 9b) [155]. The other two examples, involving STXM coupled with NEXAFS, analyze carbon materials: carbon nanotubes (CNT) (Figure 9c) and graphene oxide (GO) (Figure 9d). SXRSM analysis has been shown to reveal the electronic structure of carbon atoms in CNT coatings, exhibiting similarities to the structure of the CNTs themselves (Figure 9c) [156]. Furthermore, SXRSM enables the identification and mapping of three distinct components of carbon atom electronic structures in GO (Figure 9d) [157]. While SXRSM techniques are highly effective for characterizing electronic structures at the nanoscale, it is important to note that the chemical profiling of interfaces or interfacial phenomena is limited by the current spatial resolution—approximately 20 nm in the case of XRSP—thus making this approach less suitable for detailed interfacial studies at the atomic level.

## 4. Techniques Providing 3D Structural Information

### 4.1. APT

**Figure 10:**
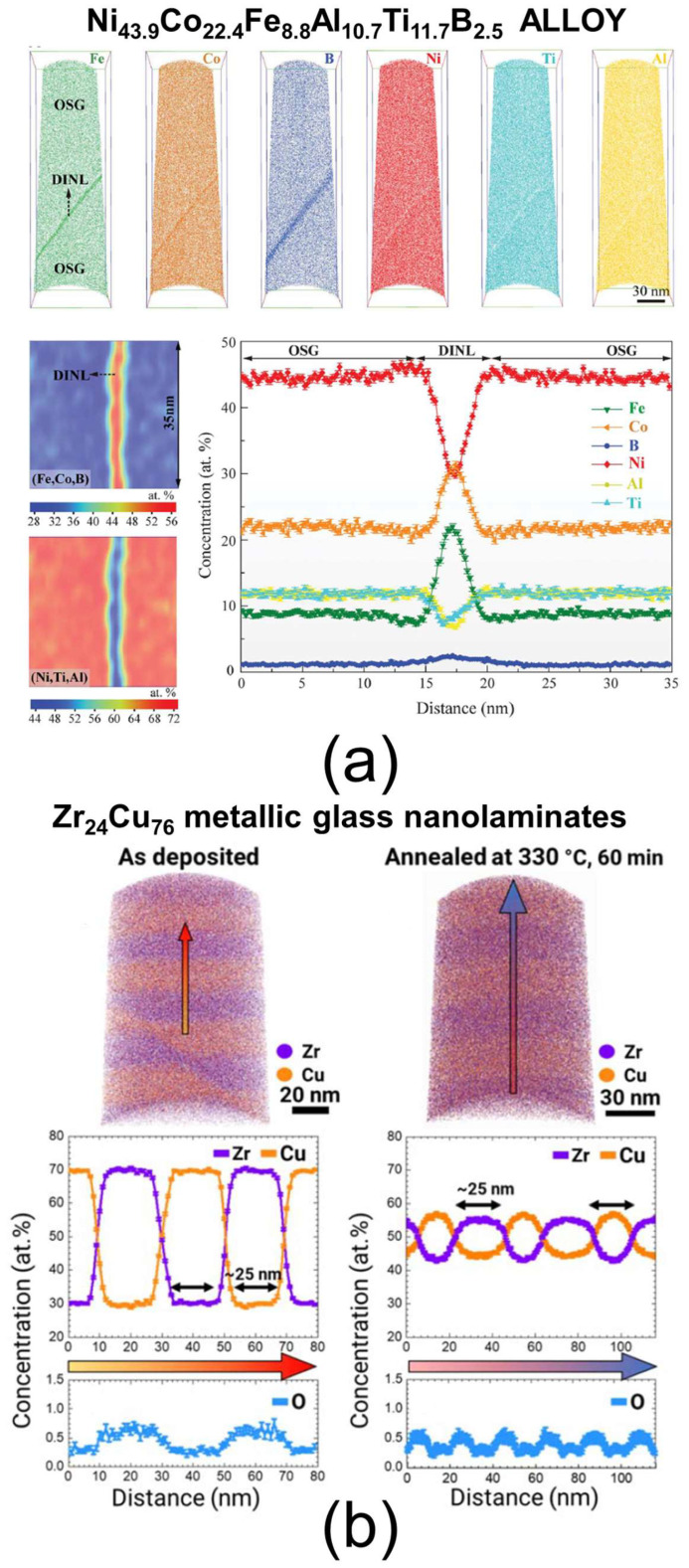
Examples of APT analyses performed in the cases of (**a**) a Ni_43.9_Co_22.4_Fe_8.8_Al_10.7_Ti_11.7_B_2.5_ alloy and (**b**) a Zr_24_Cu_76_ metallic glass nano-laminate. (**a**) shows the 3D distribution of each element in the ordered superlattice grain area (OSG) and disordered interfacial nano-layer (DINL). It is to be noted that Fe, Co, and B are enriched in the DINL area, while Ni, Al, and Ti exhibit an opposite behavior. This finding is also highlighted with a 2D compositional contour map and 1D compositional profile [162]. (**b**) represents the 3D distribution of each element before and after heat treatment, as well as a 1D compositional profile of Zr, Cu, and O crossing the successive nano-layers, highlighting a diffusion mechanism after heat exposure without oxidation [163].

Atomic probe tomography (APT) is a cutting-edge technique that delivers atomic-scale imaging and the quantification of materials. With a spatial resolution typically between 0.3 and 0.5 nm and a depth of analysis of 0.1 to 0.3 nm [158], it provides detailed three-dimensional atomic information. The reconstructed data enable the visualization of individual atoms in 3D and 2D atom maps, line scans of elemental composition, mass spectra, and precise compositional analysis. A key advantage of APT is its equal sensitivity to all elements and their isotopes, allowing the detection of elements present as interstitial diluents, as well as features such as dislocations, crystal grains, stacking faults, twins, grain boundaries, grain orientations, and segregation phenomena at interfaces. The technique relies on the field evaporation of individual atoms (ions) from a needle-shaped sample surface, followed by mass spectrometry to identify their elemental or isotopic nature and their original position, enabling 3D reconstruction. A crucial specificity of APT is that samples must be prepared in the form of a needle with an apex radius typically between 50 and 100 nm [159,160]. The standard preparation method involves the use of FIB-SEM. SEM helps identify the region of interest, while FIB enables precise fabrication of the needle-shaped specimens. Multiple APT specimens can often be prepared in a single session using this approach, with detailed procedures described in Refs. [160,161]. First, a 15–40 µm thick sample wedge is prepared and cut from the initial surface using FIB. A micromanipulator, attached via platinum deposition, lifts out this wedge from the original sample. The wedge is then aligned with a microtip array and firmly attached through platinum welding. Afterward, the wedge is sliced from the array for use in fabricating additional APT specimens. To create a needle-shaped specimen, the wedge on the microtip is subjected to an annular milling process, shaping it into a very sharp tip. Once installed in a vacuum chamber, the specimen is cooled to cryogenic temperatures to minimize atomic motions. It is then biased at a high voltage, which, combined with the small apex radius, generates a strong electrostatic field just below the surface. By applying a laser or voltage pulse, individual atoms are ionized and ejected from the tip, then projected onto a position-sensitive detector. The detector measures the ions’ time of flight (to determine their mass-to-charge ratio) and their impact position (X, Y coordinates). From the ions’ arrival order and impact positions, the system reconstructs their original spatial positions on the tip, enabling a three-dimensional atomic-scale reconstruction of the material [158]. Some examples of APT analysis on metallic-based materials are shown in Figure 10. In the first example (Figure 10a), a Ni_43.9_Co_22.4_Fe_8.8_Al_10.7_Ti_11.7_B_2.5_ alloy featuring an interfacial nano-layer is examined. The high spatial resolution of APT reveals that this nano-layer is a multi-element co-segregation of Fe, Co, and B. Specifically, the content of these three elements is increased within the nano-layer compared to the surrounding grains, while Ni, Al, and Ti show a depletion in the same region [162]. In the second example (Figure 10b), a Zr_24_Cu_76_ metallic glass nano-laminate is studied both before and after heat treatment to investigate the effects of thermal exposure on its elemental distribution. The analysis demonstrates that heat treatment induces interdiffusion between Zr and Cu without any oxidation occurring [163]. APT is an exceptionally valuable technique for obtaining three-dimensional chemical maps with sub-nanometer resolution. However, it relies on complex sample preparation processes. Additionally, a key limitation is the potential blurring of atomic positions and the deformation of inclusions during reconstruction, caused by non-uniform electrostatic fields and the physics of field evaporation. Furthermore, inaccuracies in composition measurements can arise due to correlations between the measured data and the specific experimental conditions, which must be carefully considered during data interpretation [160].

### 4.2. ET

**Figure 11:**
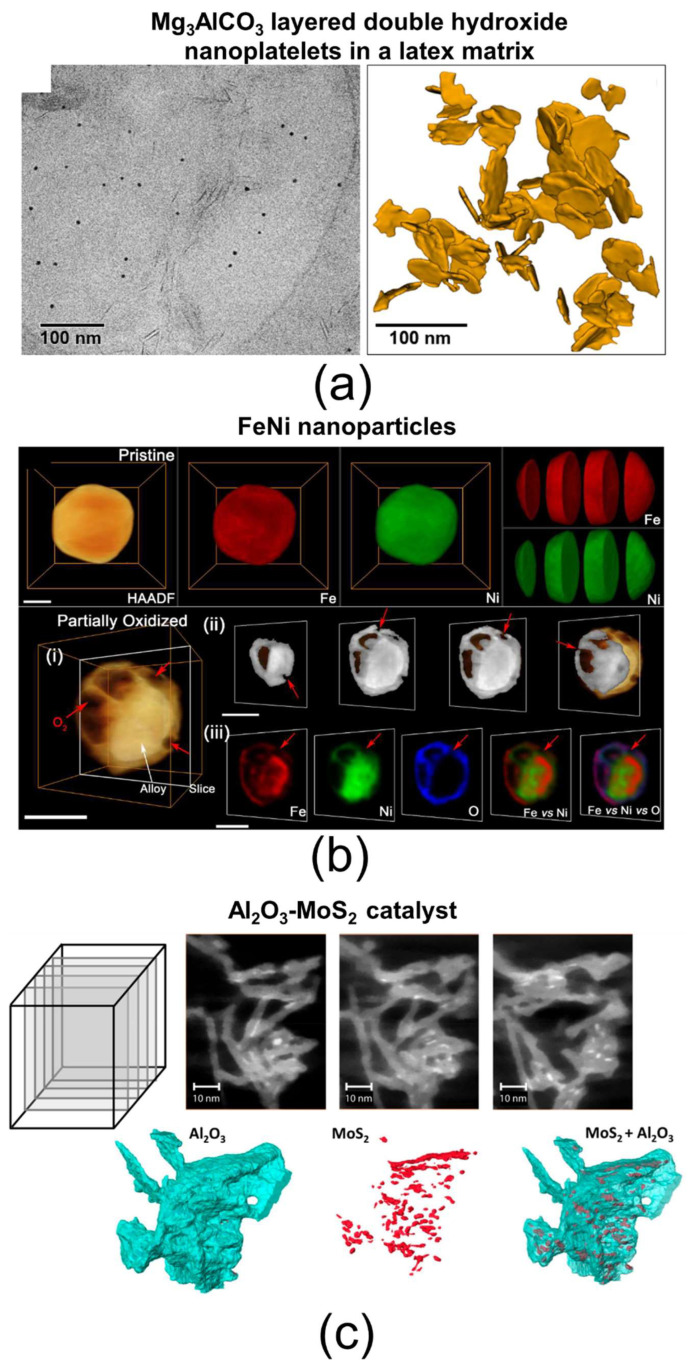
Examples of ET analysis conducted in the cases of (**a**) Mg_3_AlCO_3_ layered double hydroxide nanoplatelets dispersed within in a latex matrix, FeNi nanoparticles, and (**c**) an Al_2_O_3_-MoS_2_ catalyst. (**a**) represents a BF-TEM image of the composite and the 3D reconstruction of the nanoplatelets, highlighting their spatial distribution (reproduced with permission [172], Copyright 2019, Elsevier). (**b**) reports the 3D reconstruction of NiFe nanoparticles with identification of the different elemental phases by EDS tomography, without and with thermal oxidation. The data highlight the formation of a complex 3D structure with the formation of an oxide shell and pinholes (reproduced with permission [173], Copyright 2018, ACS). (**c**) represents slices extracted from the 3D reconstruction of the catalyst based on BF-TEM projections and the 3D reconstruction with the identification of the elemental phase obtained by EDS tomography, showing the presence of Al_2_O_3_ support particles decorated by MoS_2_ lamellar crystallites as the active phase [174].

Electron tomography (ET) has been implemented in transmission electron microscopy (TEM) to obtain three-dimensional (3D) structural information at the nanoscale from a series of two-dimensional (2D) projection images [164,165,166]. In this method, ultrathin sections are imaged over a wide tilt angle range—ideally ±90°, though practically limited to about ±70° due to shadowing effects from the specimen holder and chamber space constraints—at increments of 1–2°. Using dedicated software, these projections are reconstructed into a 3D volume with a spatial resolution reaching approximately 0.7 nm (voxel size of 0.7 nm^3^) [167]. The goal of ET is to generate a 3D representation based on mass contrast, which depends on the thickness of the phase crossed by the electron beam. To prevent diffraction effects that could distort this contrast, the anisotropic dark-field (ADF) detector is used to select electron scattering dominated by phonon interactions, enhancing mass–thickness contrast [168]. Some ET techniques also exploit diffraction contrast—using special conditions to record dark-field (DF) images—to reveal specific structural features such as crystalline orientation and dislocations [169]. Furthermore, 3D reconstruction can be extended to elemental mapping images obtained via energy-dispersive X-ray spectroscopy (EDS) or electron energy-loss spectroscopy (EELS), following the same principles as projection-based reconstructions, achieving voxel sizes around 1 nm^3^ [170,171]. Combining mass–thickness contrast with 3D elemental distribution provides new opportunities to detect structures impossible to resolve with 2D techniques. The quantitative analysis of the reconstructed objects—such as size, shape, and orientation—is enabled by dedicated software, which also allows for advanced 3D visualization. Several examples of ET analysis are shown in Figure 11. In Figure 11a, the 3D reconstruction visualizes Mg_3_AlCO_3_ layered double hydroxide nanoplatelets dispersed within a latex matrix, allowing the assessment of their spatial distribution within the polymer [172]. In Figure 11b, EDS tomography provides insights into the oxidation mechanisms of NiFe nanoparticles, revealing the formation of pinholes and oxide shells [173]. In Figure 11c, EDS tomography visualizes the distribution of MoS_2_ catalyst active particles on an Al_2_O_3_ support, facilitating an evaluation of catalyst quality [174].

### 4.3. PXCT

**Figure 12:**
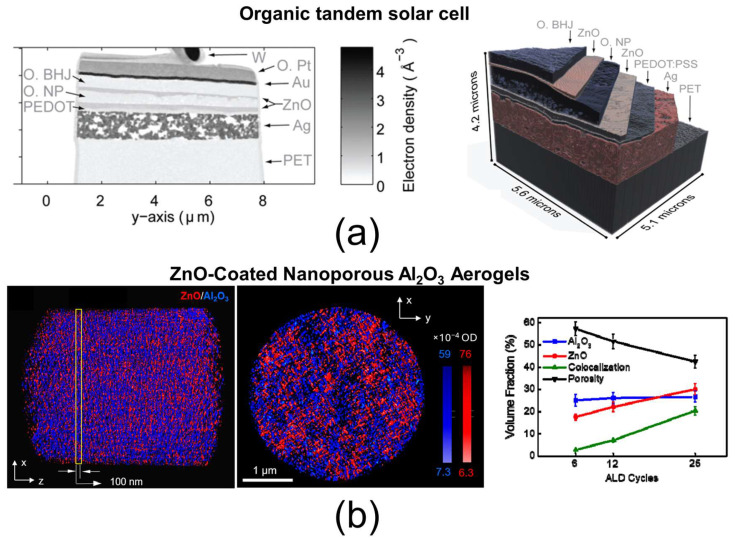
Examples of PXCT analysis performed in the cases of (**a**) an organic tandem solar cell and (**b**) a ZnO-coated nanoporous Al_2_O_3_ aerogel. (**a**) represents a 2D slice of the reconstructed volume and the reconstructed volume showing the different layers of the solar cell (reproduced with permission [178], Copyright 2015, RSC). (**b**) shows the reconstructed volume with the two material phases, as well as the evolution of structural parameters measured by PXCT (volume fraction of each phase, porosity, and colocalization) as a function of the amount of atomic layer deposition (ALD) [179].

Ptychographic X-ray computed tomography (PXCT) is a recent technique developed at synchrotron facilities that enables the 3D reconstruction of a sample based on mass–thickness contrast from a series of 2D diffraction projections. These projections are diffraction patterns acquired as the sample is rotated in steps of typically 1°, over a total rotation of about 180°, with the diffraction patterns recorded in two perpendicular directions. Unlike conventional X-ray imaging methods limited by lens resolution, PXCT leverages coherent diffraction imaging (CDI) principles; the diffraction patterns are mathematically phased and inverted to produce lensless images, which are then used for the 3D reconstruction of the sample [175,176,177]. A spatial resolution of approximately 16 nm (voxel size of 16 nm^3^) has been reported for PXCT [175]. The samples analyzed are generally micrometer-sized cones or cylinders, often prepared via FIB milling. Two examples of PXCT measurements are shown in Figure 12: (a) an organic tandem solar cell [178] and (b) a ZnO-coated nanoporous Al_2_O_3_ aerogel [179]. In both cases, the 3D reconstructions have been segmented into different material phases, allowing for quantitative analysis—such as tracking how the volume fraction of each phase evolves with various treatments (Figure 12b). While PXCT is highly attractive for scanning large volumes with nanometric resolution, it does have notable limitations. These include complex sample preparation procedures, intricate data processing workflows, and the limited availability of the technique to a few research centers with synchrotron access. Additionally, PXCT based solely on mass–thickness contrast cannot discern the chemical composition of phases; thus, it often needs to be combined with chemical analysis techniques to fully characterize the sample’s chemical nature.

Finally, in cases where techniques capable of providing comprehensive 3D structural and chemical composition data are not available, an initial approach involves combining 2D imaging techniques with sputtering methods to acquire in-depth profiling information at nanometer resolution. Examples of such methods include AES coupled with ion sputtering [124,180] and nanoSIMS [131,181].

## 5. Emerging Techniques and Methods

It is important to recognize that the characterization techniques and methods discussed in this review are continually evolving, driven by machine manufacturers, research laboratory specialists, and, in the case of synchrotron-based techniques, by beamline scientists. In this section, we briefly present several noteworthy advancements in characterization techniques that are especially relevant for investigating nanolayered materials.

The resolution of X-ray tomography at synchrotron facilities has significantly improved, thanks to advancements such as ptychographic X-ray computed tomography—particularly with the implementation of burst ptychography mode—which now enables resolutions of approximately 7 nm [182,183]. This emerging technique is expected to have a transformative impact on the fields of materials science and engineering, as well as biology [183]. Similarly, soft X-ray spectromicroscopy (SXRSM) has recently been enhanced through the development of X-ray spectro-ptychography (XRSP) [143]. These advancements involve the use of newly developed cameras sensitive to low-energy X-rays [184]. As a result, XRSP measurements can now be conducted at low photon energies—such as the carbon K-edge (285 eV) and lithium K-edge (55 eV)—which is particularly relevant for the characterization of organic electronic materials and lithium-ion batteries.

Additionally, AFM-IR technology continues to advance [185], with the latest laboratory systems achieving lateral resolutions of approximately 10 nm and improved signal-to-noise ratios [186,187]. These developments enable the chemical mapping of thick nano-layers, making the technique highly relevant for the characterization of such materials. Recent innovations in AFM-IR include the integration of surface and depth-probing analyses to achieve the tomographic imaging of materials [188], as well as multimodal imaging combined, for example, with the nanomechanical mode [189].

Correlative microscopy, which involves the use of multiple microscopy techniques to analyze the same material area, is gaining increasing interest in the study of nanomaterials [190,191]. This approach provides complementary structural information, leading to a better understanding of material formation and performance. All the microscopy techniques cited in this review can, in theory, be combined to analyze the same nano-layer areas. The main limitations are that the techniques must be compatible with the same sample preparation method (Table 3), and that each technique used in sequence must not damage the targeted region of interest.

It is also important to note that the use of machine learning algorithms for image and spectral analysis is gaining significant attention in materials characterization. These approaches can process large volumes of data, enhance the signal-to-noise ratio, facilitate the recognition of structural features within datasets, and even reveal hidden structural information [192,193]. However, a key challenge lies in preserving the physical integrity and underlying meaning of the data during the successive application of computational corrections.

These technical advancements are likely to reveal previously undiscovered structural properties of nanolayers. Consequently, regularly reviewing and updating the capabilities and performance of available characterization techniques and methods is crucial to avoid overlooking advanced techniques that could provide critical insights into nano-layer analysis.

## 6. Conclusions

This review aimed to summarize the techniques available for obtaining structural information on nano-AdLs and nano-RLs within material assemblies. It serves as a practical guide for selecting the most appropriate method based on the specific targeted property, including considerations for sample preparation. The advantages and limitations of each technique are briefly described to assist in designing an effective characterization strategy tailored to these complex material systems.

To accurately characterize a nano-layer, it is crucial to select techniques and methods with a lateral resolution finer than the size of the feature of interest. The greater the difference, the more detailed the analysis, enabling effective profiling and mapping. Once the appropriate technique is chosen, careful attention must be paid to sample preparation, as it significantly impacts data quality. The main preparation methods include fracturing or cutting, mechanical polishing, ultramicrotomy, ion beam milling, and focused ion beam (FIB) milling. Consulting with a sample preparation expert is highly recommended, especially for advanced techniques such as ultramicrotomy and FIB, which typically involve multiple steps. Achieving an optimal sample shape, dimensions, surface roughness, and site-specific region of interest (nano-layers) is challenging. Moreover, it is essential to consider whether observed features reflect the intrinsic properties of the nano-layers or are artifacts introduced during preparation. The most robust structural insights often come from combining different techniques—such as TEM coupled with EDS, EELS, or EFTEM; or SXRSM, TERS, and tomography methods like ET—allowing for the correlation of morphological and chemical data in both two and three dimensions. Employing tomography enables detailed 3D nanoscale structural information, integrating morphology with chemical composition, which is highly valuable for understanding complex material systems. It is also advisable to cross-verify data using different techniques due to the inherent limitations and potential errors of each method. This approach is particularly important when investigating interfacial phenomena based on elemental composition, where experimental artifacts can lead to misinterpretations.

A notable observation is that nano-AdL and nano-RL systems have primarily been investigated using SEM, TEM, and AFM, with limited data available from other nanoscale characterization techniques. To gain a more comprehensive understanding of their initial structure and long-term durability, efforts should be directed toward extending the scope of analysis by employing additional techniques. For instance, methods that simultaneously provide morphological and chemical information—such as TEM combined with EDS, EELS, electron tomography (ET), TERS, or SXRSM/XRSP—would be highly relevant. However, meticulous attention must be given to sample preparation to avoid compromising the assembly’s integrity. This preparation step can be particularly challenging, especially in the case of nano-RLs, where maintaining the interface and structural integrity is complex yet essential for reliable characterization.

Most of the characterization techniques discussed in this review have the capability to expose the sample to stimuli such as heat, stress, or humidity, enabling in situ analysis. This is especially valuable for techniques providing 3D structural information, as it opens the possibility of acquiring 4D data in order to capture temporal evolution of structures at the nanoscale. A dedicated review focusing on in situ techniques and methods for investigating material durability and functionality at the nanoscale could serve as the subject of a future publication.

## Figures and Tables

**Table 1:** Summary of techniques for determining the structural parameters of nano-AdLs and nano-RLs (including related sections for easy reference).

Techniques	Typical Spatial Resolution *	Structural Information	Section
Scanning electron microscopy (SEM) + energy-dispersive X-ray spectrometry (EDS)	1 nm for morphological imaging	Morphological imaging, thickness of the layer, layer homogeneity and bonding quality (qualitative) by scanning large samples, element presence (qualitative)	3.1
Transmission electron microscopy (TEM) + Electron nanodiffraction (END) + energy-dispersive X-ray spectrometry (EDS) + electron energy loss spectroscopy (EELS)	0.1 nm for morphological imaging 1 nm–20 nm for chemical analysis	Morphological imaging, thickness of the layer and interfacial region, 2D elemental composition/profiling/mapping, elemental bonding, crystal structure and defects, interfacial region chemistry and morphology	3.2
Atomic force microscopy (AFM) AFM + nanomechanical mode Conductive AFM (c-AFM) AFM + infrared spectroscopy (AFM-IR)	1 nm–15 nm for AFM, AFM + nanomechanical mode, and c-AFM 20 nm–100 nm for AFM-IR	Morphological imaging, layer thickness, layer homogeneity and bonding quality (qualitative) by scanning large samples, interfacial region morphology, viscoelastic properties of the layer and interfacial region, electrical conductivity of the layer and interfacial region, identifying a material thanks to the recorded IR spectrum (qualitative), verifying the presence or not of a given chemical bond in the layer (qualitative)	3.3
Scanning helium ion microscopy (HIM)	0.5 nm	Morphological imaging of the layer and interfacial region, thickness of the layer, layer homogeneity and bonding quality (qualitative) by scanning large samples, ionoluminescence, crystallographic texture	3.4
Auger electron spectroscopy (AES)/scanning Auger microscope (SAM)	10 nm	Two-dimensional elemental composition/profiling/mapping, in-depth elemental profiling if combined with an ions milling sputtering method	3.5
Nanoscale secondary ion mass spectrometry (nanoSIMS)	30 nm	Two-dimensional elemental composition/profiling/mapping, in-depth elemental profiling, including isotope analysis, detection of trace elements	3.6
Tip-enhanced Raman spectroscopy (TERS)	10 nm–50 nm	Topographic imaging and 2D chemical composition/profiling/mapping based on Raman spectroscopy (including crystalline phase identification, and crystallinity assessment), information about structural rearrangements during the application of stress and/or heat	3.7
Soft X-ray spectromicroscopy (SXRSM)/X-ray spectro-ptychography (XRSP)	10 nm–100 nm for morphological imaging with SXRSM, 3 nm for morphological imaging with XRSP 30 nm for chemical analysis with SXRSM, 20 nm for chemical analysis with XRSP	Absorption and phase-contrast morphological imaging, for a given atom the oxidation state/coordination chemistry, neighbor distances/coordination number/species, the electronic structure is sensitive to spatial orientation (induced by the application of stress)	3.8
Atom probe tomography (APT)	0.3–0.5 nm	Three-dimensional elemental composition, including mapping and profiling, interfacial region chemistry	4.1
Electron tomography (ET)	0.7 nm for mass–thickness imaging 1 nm for chemical analysis	Three-dimensional mass–thickness imaging, three-dimensional elemental composition, including mapping and profiling, layer thickness, interfacial region morphology and chemistry	4.2
X-ray ptychographic computed tomography (PXCT)	16 nm	Three-dimensional mass–thickness imaging, layer thickness	4.3

* It is important to note that the maximum resolution indicated in this table is generally a theoretical value. Achieving this resolution requires the use of the most advanced equipment, specific operating conditions, simplified sample formulations, optimal sample preparation, and high sample stability throughout the measurement process.

**Table 2:** Summary of structural parameters of nano-AdLs and nano-RLs and corresponding techniques.

Structural Parameter	Technique
2D morphological/topographical imaging	SEM, TEM, AFM, HIM, TERS, SXRSM/XRSP
3D morphological imaging	ET, PXCT
Thickness of the layer	SEM, TEM, AFM, HIM, ET, PXCT
Homogeneity of the layer by scanning large samples	SEM, AFM, HIM
Bonding quality (qualitative) by scanning large samples	SEM, AFM, HIM
Element presence (qualitative)	SEM+EDS
Material identification (qualitative)	AFM-IR
2D elemental composition/profiling/mapping	TEM+EDS, TEM+EELS, AES/SAM, nanoSIMS, TERS, APT, ET
3D elemental composition	APT, ET
In-depth elemental profiling	AES/SAM + ion milling sputtering, nanoSIMS
Detection of trace elements	nanoSIMS
Elemental bonding (qualitative)	AFM-IR
Elemental bonding	TEM+EELS
Crystal structure and defects	TEM+END
Interfacial region morphology	TEM, AFM, HIM, ET
Interfacial region chemistry	TEM+EDS, TEM+EELS, APT, ET
Interfacial region viscoelastic properties	AFM + nanomechanical mode
Viscoelastic properties of the layer	AFM + nanomechanical mode
Electrical conductivity of the layer	c-AFM
Interfacial region electrical conductivity	c-AFM
Crystallographic texture	HIM
Ionoluminescence	HIM
Crystalline phase identification, crystallinity calculation	TERS
Structural rearrangements during the application of stress and/or heat	TERS, SXRSM/XRSP
For a given atom: oxidation state/coordination chemistry	SXRSM/XRSP
For a given atom: neighbor distances/coordination number/species	SXRSM/XRSP

**Table 3:** Methods for preparing the cross-section of a material assembly.

Method	Fracturing or Cutting	Mechanical Polishing	Ultramicrotome (UM) Planarization or Thin/Ultrathin Sectioning	Ion Beam (IB) Milling and Polishing	Focused Ion Beam (FIB) Milling and Polishing
Sample area/volume	Overall sample section.	Polished area: few mm^2^ to few tens of mm^2^.	Planarized area: <few mm^2^. Thin section: few mm × few mm × submicron thickness. Ultrathin section: <0.1 mm^2^ × <100 nm (thickness).	Eroded volume: few hundreds of µm × few hundreds of µm × few tens of µm (thickness). Ultrathin section: few mm × few mm × <100 nm (thickness) at the center.	Eroded area: few tens of µm × few µm. Thin section: few µm × few mm × submicron to few µm for the thickness. Ultrathin section: few tens of µm × few µm × <100 nm (thickness). Needle-shaped sample: tip radius at the apex in the range of 50 nm–150 nm, length in the range of few µm to few tens of µm. Cone/cylinder-shaped sample: maximum diameter < 6 µm, height < 10 µm.
Advantages	This method is fast, very easy to implement, and cost-effective, making it ideal for quickly visualizing the overall sample cross-section.	This is an easy and cost-effective method that does not require advanced technical expertise. Mechanical polishing can be combined with chemical etching to further reveal the material’s internal structure or with chemical polishing to remove residual scratches and/or weaken the bonds between surface atoms. This enhances the removal of surface material during subsequent mechanical polishing. Typically, mechanical polishing serves as a preparatory step for ion beam milling or FIB, helping to accelerate the overall sample preparation process. Depending on specific needs, various systems can be employed, such as a precision disk grinder (for fine mechanical polishing with micrometer steps), a tripod polisher (for reducing sample thickness to electron transparency), or a dimple grinder (for thinned central areas of the sample).	This method allows for sample preparation in a clean environment, minimizing contamination. Recent advancements include the integration of an ultramicrotome stage within an SEM, enabling in situ imaging during sample planarization. This facilitates in-depth imaging of the cross-section with enhanced precision.	There is no pressure applied to the sample, thereby avoiding the introduction of mechanical artifacts. Ion beam (IB) milling can also be employed to effectively remove surface artifacts produced during FIB milling, resulting in a cleaner, more accurate surface.	FIB is particularly useful when precise positioning below the micron scale or specific orientation for milling is required within a sample. Since there is no pressure applied to the sample, this method avoids mechanical artifacts often associated with other preparation techniques. Multiple milling steps, typically in the order of 0.1 µm, can be performed to analyze the structure as a function of cross-section depth. When combined with an SEM column (FIB-SEM), the system allows for the integration of additional instruments, such as an atomic force microscope (AFM) head or a secondary ion mass spectrometer (SIMS), enabling in situ correlative analyses. Additionally, FIB can be used to deposit materials on the sample surface through the dissociation of precursor gases, allowing for various site-specific modifications or enhancements.
Drawbacks	Applying pressure to break the sample can induce artifacts such as local plastic deformation, damage, and debris. The fracture or cut path is difficult to control, resulting in low reproducibility and a very rough surface. Consequently, interpreting the structure becomes complex, and elemental analysis is less reliable due to the need for a very flat, smooth surface.	The application of pressure during sample preparation may induce artifacts. Additionally, performing multiple steps to finely study the structure as a function of cross-section depth is challenging due to significant material loss during polishing.	A high level of expertise is required to effectively utilize this method. It also necessitates an initial trimming step, during which the exact position of the region of interest must be precisely known and controlled. The diamond knives used to cut hard materials are very expensive to replace if damaged; however, they can often be resharpened if damage is limited. This method may induce local deformation or damage to the sample due to the applied compression by the knife. Additionally, ultrathin sections cannot be obtained from very hard, very soft, very brittle, or highly heterogeneous samples using this technique.	Due to the limited milling depth, the region of interest must be located near the edge of the sample. To address this, areas outside the region of interest can be removed through mechanical polishing. For ultrathin section preparation, a pre-thinning step—using techniques such as mechanical polishing, magnetorheological polishing, or dimpling—is typically performed. The final thinning with an ion beam (IB) is usually carried out on the opposite surface of the pre-thinned area. It is important to note that ion beam milling can induce surface amorphization and alter the surface topography of the sample. Additionally, IB milling is generally not suitable for isolating regions of interest below the micron scale.	Only small volumes can be prepared using this method. In the case of electrically insulating samples, charging effects may occur due to the positive ion beam, leading to deflection of the beam and potential inaccuracies. For thermally insulating materials, the ion beam can generate local heating, causing damage; using cryogenic FIB is often suitable to mitigate this issue. FIB milling can induce surface artifacts such as amorphization, Ga implantation, lattice strains, and crystal defects, with a higher production of artifacts compared to ion beam (IB) techniques. Due to limited milling depth, the region of interest must be close to the edge of the sample. To address this, the non-interesting areas can be removed through mechanical polishing. Additionally, to decrease the milling time needed to prepare ultrathin sections, pre-thinning of the sample via mechanical methods is often employed.
Targeted characterization techniques	SEM, AES/SAM, HIM	SEM, AES/SAM, AFM, nanoSIMS, HIM	Planarized area: SEM (+EDS), AES/SAM, AFM, SIMS, HIM. Thin section: nanoSIMS, SEM (+EDS), AES/SAM, AFM, HIM. Ultrathin section: SEM (+EDS), AES/SAM, AFM, nanoSIMS. TEM/ET (+END+EDS+EELS), TERS, SXRSM/XRSP.	Eroded volume: SEM (+EDS), AES/SAM, AFM, SIMS, HIM. Ultrathin section: SEM (+EDS), AES/SAM, AFM, nanoSIMS, TEM/ET (+END+EDS+EELS), TERS, SXRSM/XRSP.	Eroded area: SEM (+EDS), AES/SAM, AFM, SIMS, HIM. Thin section: nanoSIMS, SEM (+EDS), AES/SAM, AFM, HIM. Ultrathin section: SEM (+EDS), AES/SAM, AFM, nanoSIMS, TEM/ET (+END, +EDS+EELS), TERS, SXRSM/XRSP. Needle-shaped sample: APT Cone/cylinder-shaped sample: PXCT.

## Data Availability

No new data were generated or analyzed in this study; therefore, data sharing is not applicable.

## References

[1] Gay C. (2002). Stickiness: Some Fundamentals of Adhesion. Integr. Comp. Biol..

[2] Mo Z., Wang F., Li J., Liu Q., Zhang G., Li W., Yang C., Sun R. (2023). Temporary Bonding and Debonding in Advanced Packaging: Recent Progress and Applications. Electronics.

[3] Jong Ho R., Chang Yol Y., Joung Ah K. (2013). Copper Foil Attached to the Carrier Foil, a Method for Preparing the Same and Printed Circuit Board Using the Same.

[4] Montméat P., Enot T., Enyedi G., Pellat M., Thooris J., Fournel F. (2018). Development and adhesion characterization of a silicon wafer for temporary bonding. Int. J. Adhes. Adhes..

[5] Puligadda R., Pillalamarri S., Hong W., Brubaker C., Wimplinger M., Pargfrieder S. (2006). High-Performance Temporary Adhesives for Wafer Bonding Applications. MRS Proc..

[6] Strepenne F. (2010). Adhesion of Thin Films on Steel and Separation of the Energy Contributions. Ph.D. Thesis.

[7] Beom W.J., Lee S.H., Choi E.S., Song K.D., Kim H.C. (2018). Carrier-Foil-Attached Ultra-Thin Copper Foil.

[8] Kaidi Z., Devahif T., Kersten A., Streel M. (2023). Composite Copper Foil and Method of Fabricating the Same.

[9] Guo L., Liu J., Xia H., Li X., Zhang X., Yang H. (2021). Effects of Surface Treatment and Adhesive Thickness on the Shear Strength of Precision Bonded Joints. Polym. Test..

[10] Gleich D.M., Van Tooren M.J.L., Beukers A. (2001). Analysis and evaluation of bondline thickness effects on failure load in adhesively bonded structures. J. Adhes. Sci. Technol..

[11] Da Silva L.F.M., Rodrigues T.N.S.S., Figueiredo M.A.V., De Moura M.F.S.F., Chousal J.A.G. (2006). Effect of Adhesive Type and Thickness on the Lap Shear Strength. J. Adhes..

[12] Carbas R.J.C., Dantas M.A., Marques E.A.S., Da Silva L.F.M. (2021). Effect of the adhesive thickness on butt adhesive joints under torsional loads. J. Adv. Join. Process..

[13] Kumar S., Gaire B., Persson B.N.J., Dhinojwala A. (2024). Impact of Nanometer-Thin Stiff Layer on Adhesion to Rough Surfaces. Phys. Rev. Res..

[14] Kim K., Jeong J., Sim Y., Lee E. (2006). Minimization of residual layer thickness by using the optimized dispensing method in S-FILTM process. Microelectron. Eng..

[15] https://scifinder-n.cas.org/.

[16] Zhang Z., Wang C., Wang X., Reddy K.M., Liu P., Wang Y., Song S. (2023). Precise quantification of the adhesion between metallic thin films and silicon wafer. Thin Solid Film..

[17] Matsumae T., Kurashima Y., Takagi H., Shirayanagi Y., Hiza S., Nishimura K., Higurashi E. (2022). Room temperature bonding of GaN and diamond substrates via atomic layer. Scr. Mater..

[18] Suga T. (2006). Cu-Cu Room Temperature Bonding-Current Status of Surface Activated Bonding (SAB). ECS Trans..

[19] Liu Y., Wang G., Chen Y., Kang Q., Luo S., Li Z., Xu X., Liu Q., Sui X. (2022). Air atmosphere diffusion bonding of Al–Mg–Li alloy using Cu nano-coating interlayer: Microstructural characterization and formation mechanisms. Mater. Des..

[20] Chu W.H., Chin R., Huen T., Ferrari M. (1999). Silicon membrane nanofilters from sacrificial oxide removal. J. Microelectromechanical Syst..

[21] Lee T.-H., Huang C.-H., Yang Y.Y., Suryasindhu T., Li P.W. (2007). Nanoscale thick layer transfer of hydrogen-implanted wafer by using polycrystalline silicon sacrificial layer. Appl. Phys. Lett..

[22] Stelling C., Retsch M. (2018). Nanomeshes at Liquid Interfaces: From Free-Standing Hole Arrays toward Metal–Insulator–Metal Architectures. Adv. Mater. Interfaces.

[23] Hein E. (2011). Lithography-free glass surface modification by self-masking during dry etching. J. Nanophotonics.

[24] Liao P.-Y., Khot K., Alajlouni S., Snure M., Noh J., Si M., Zhang Z., Shakouri A., Ruan X., Ye P.D. (2023). Alleviation of Self-Heating Effect in Top-Gated Ultrathin In 2 O 3 FETs Using a Thermal Adhesion Layer. IEEE Trans. Electron. Devices.

[25] Kim A., Ahn Y., Li W., Park S.H., Cho M.J., Choi D.H., Yang H. (2023). Stretchable Semiconducting Polymers with Hydrogen-Bonding-Capable Conjugation Breakers: Synthesis and Application in Organic Thin-Film Transistors. ACS Appl. Mater. Interfaces.

[26] Montméat P., Dechamp J., Enyedi G., Fournel F., Zavvou Z., Jousseaume V. (2022). Initiated Chemical Vapor Deposition of polysiloxane as adhesive nanolayer for silicon wafer bonding. Mater. Sci. Semicond. Process..

[27] Sutjianto J.G., Yoo S.H., Westerman C.R., Jackson T.N., Wilker J.J., Gomez E.D. (2023). Blends of Conjugated and Adhesive Polymers for Sticky Organic Thin-Film Transistors. Adv. Electron. Mater..

[28] Hao L., Cheng H., Ouyang J., Huan Y., Yan J. (2022). Integration of Ferroelectric K0.5Na0.5NbO3 films on Si at 400 °C. Mater. Today Commun..

[29] Takakura R., Murakami S., Watanabe K., Takigawa R. (2023). Room-temperature bonding of Al_2_O_3_ thin films deposited using atomic layer deposition. Sci. Rep..

[30] Xu Z., He Z., Yuan Y.J. (2018). Investigation of sacrificial layer masking fabrication of dual-frequency quartz crystal microbalance. Sens. Actuators A Phys..

[31] Yasuda S., Miyagawa T., Yonezu A., Ishibashi K. (2023). Laser shock-wave adhesion test (LaSAT) and ab initio calculations for adhesive strength evaluation of thin metallic films. Mater. Today Commun..

[32] Amino T., Uomoto M., Shimatsu T. (2022). Bonding performance in atomic diffusion bonding of wafers using amorphous Si thin films with smooth surface. Jpn. J. Appl. Phys..

[33] Matsui J., Kubota K., Kado Y., Miyashita T. (2007). Electroless Copper Plating onto Polyimide Using Polymer Nanosheet as a Nano-Adhesive. Polym. J..

[34] Toru H., Akitoshi T., Tetsuhiro M., Ayumu T. (2016). Ultrathin Copper Foil with Carrier and Method for Manufacturing Same.

[35] Zhang X., Zhang P., Zhang W., Chen J., Hu F. (2023). Preparation of UV Curable Optical Adhesive NOA81 Bionic Lotus Leaf Structure Films by Nanoimprint Technique and the Applications on Silicon Solar Cells. Coatings.

[36] Li X., Yan X., Zhao Z., Mohamed M.S., Wang J., Zhu X. (2021). FDTD simulation on transmittance of silica microsphere thin films with varying embedding in an optical adhesive. Optik.

[37] She S., Zhang Y., Huang Z., Zhou J., Ke S. (2022). Effect of the Thickness of the a-Si Bonding Layer at InGaAs/Si Bonded Interface on the Performance of InGaAs/Si Avalanche Photodiode. Acta Photonica Sin..

[38] Zhang X., Liu X., Liu L., Han Y., Tan H., Liu L., Lin Z., Yu S., Wang R., Cai X. (2023). Heterogeneous integration of III–V semiconductor lasers on thin-film lithium niobite platform by wafer bonding. Appl. Phys. Lett..

[39] Yamada Y., Fukumoto S., Fujimoto K. (2021). Mechanism for Adhesion of Epoxy Resin to Copper Substrate through C-H-Si Amorphous Thin Film. J. Smart Process..

[40] Li J., Zhu P., Liu L., Huang Y. (2023). Preparation Method of Nano Conductive Adhesive.

[41] Howlader M.M.R., Zhang F., Deen M.J., Suga T., Yamauchi A. (2011). Surface activated bonding of copper through silicon vias and gold stud bumps at room temperature. J. Vac. Sci. Technol. A Vac. Surf. Film..

[42] Chua S.T., Siow K.S. (2016). Microstructural studies and bonding strength of pressureless sintered nano-silver joints on silver, direct bond copper (DBC) and copper substrates aged at 300 °C. J. Alloys Compd..

[43] Tan S., Xiang R. (2015). Thermally-Conductive Adhesive with High Thermal Conductivity and High Ageing Resistance as well as Preparation Method and Application Thereof.

[44] Ding Y., Garland S., Howland M., Revzin A., Pan T. (2011). Universal Nanopatternable Interfacial Bonding. Adv. Mater..

[45] Ding Y., Garland S., Howland M., Revzin A., Pan T. (2013). Universal Anisotropically Conductive Nano-adhesive of PDMS Oligomers. MRS Proc..

[46] Marques A., Mocanu A., Tomić N., Balos S., Stammen E., Lundevall A., Abrahami S.T., Günther R., de Kok J.M.M., de Freitas S.T. (2020). Review on Adhesives and Surface Treatments for Structural Applications: Recent Developments on Sustainability and Implementation for Metal and Composite Substrates. Materials.

[47] Şakalak H., Yilmaz K., Gürsoy M., Karaman M. (2022). Roll-to-Roll Vapor Deposition of Hydrophobic and Transparent Nano-Adhesive Polymeric Thin Films on Rigid and Flexible Substrates. Ind. Eng. Chem. Res..

[48] Hashemi Nasr F., Barikani M., Salehirad M. (2018). Preparation of self-healing polyurethane/functionalized graphene nanocomposites as electro-conductive one part adhesives. RSC Adv..

[49] Chen J., Liu J., Thundat T., Zeng H. (2019). Polypyrrole-Doped Conductive Supramolecular Elastomer with Stretchability, Rapid Self-Healing, and Adhesive Property for Flexible Electronic Sensors. ACS Appl. Mater. Interfaces.

[50] Liu H. (2015). Nano superpolymer high-flame-retardancy anti-aging adhesive and preparation method thereof.

[51] Mourdikoudis S., Pallares R.M., Thanh N.T.K. (2018). Characterization techniques for nanoparticles: Comparison and complementarity upon studying nanoparticle properties. Nanoscale.

[52] Sikora M., Wojcieszak D., Chudzyńska A., Zięba A. (2023). Improved Methodology of Cross-Sectional SEM Analysis of Thin-Film Multilayers Prepared by Magnetron Sputtering. Coatings.

[53] Höflinger G. (2014). Brief Introduction to Freeze Fracture and Etching. https://www.leica-microsystems.com/science-lab/life-science/brief-introduction-to-freeze-fracture-and-etching/.

[54] Li X., Mu Z., Song X. (2024). Microstructure and Stress Evolution of W Nanofilms Prepared by Arc Ion Plating under Different Deposition Time and Substrate Bias. J. Mater. Sci..

[55] Jebali S., Vayer M., Belal K., Mahut F., Sinturel C. (2024). Dip-Coating Deposition of Nanocomposite Thin Films Based on Water-Soluble Polymer and Silica Nanoparticles. Colloids Surf. A Physicochem. Eng. Asp..

[56] Buzea C., Beydaghyan G., Elliott C., Robbie K. (2005). Control of Power Law Scaling in the Growth of Silicon Nanocolumn Pseudo-Regular Arrays Deposited by Glancing Angle Deposition. Nanotechnology.

[57] Panjan P., Drnovšek A., Dražić G. (2021). Influence of Growth Defects on the Oxidation Resistance of Sputter-Deposited TiAlN Hard Coatings. Coatings.

[58] Hayles M.F., De Winter D.A.M. (2021). An Introduction to cryo-FIB-SEM Cross-sectioning of Frozen, Hydrated Life Science Samples. J. Microsc..

[59] Biddut A.Q., Zhang L.C., Ali Y.M., Liu Z. (2008). Damage-Free Polishing of Monocrystalline Silicon Wafers without Chemical Additives. Scr. Mater..

[60] Vlcak P., Jirka I. (2016). Protective Sliding Carbon-Based Nanolayers Prepared by Argon or Nitrogen Ion-Beam Assisted Deposition on Ti6Al4V Alloy. J. Nanomater..

[61] Kim S., Bark C.W. (2020). Effect of Surface Treatment by Chemical-Mechanical Polishing for Transparent Electrode of Perovskite Solar Cells. Energies.

[62] Samim P.M., Fattah-alhosseini A., Elmkhah H., Imantalab O. (2021). Nanoscale Architecture of ZrN/CrN Coatings: Microstructure, Composition, Mechanical Properties and Electrochemical Behavior. J. Mater. Res. Technol..

[63] Cairney J.M., Munroe P.R., Hoffman M. (2005). The Application of Focused Ion Beam Technology to the Characterization of Coatings. Surf. Coat. Technol..

[64] Keim E.G., Bijker M.D., Lodder J.C. (2001). Preparation of Cross-Sectional Transmission Electron Microscopy Specimens of Obliquely Deposited Magnetic Thin Films on a Flexible Tape. J. Vac. Sci. Technol. A Vac. Surf. Film..

[65] Cha H.-W., Kang M.-C., Shin K., Yang C.-W. (2016). Transmission Electron Microscopy Specimen Preparation of Delicate Materials Using Tripod Polisher. Appl. Microsc..

[66] Enganati S.K., Addiego F., Fernandes J.P.C., Koutsawa Y., Zielinski B., Ruch D., Mertz G. (2021). Multiscale Characterization of the Interfacial Region in Flexible Rubber Composites: Initial Structure and Evolution upon Thermal Treatment. Polym. Test..

[67] Ranner R., DeRose J. (2019). Introduction to Ultramicrotomy. https://www.leica-microsystems.com/science-lab/life-science/introduction-to-ultramicrotomy/.

[68] Zhang H., Wang C., Zhou G. (2020). Ultra-Microtome for the Preparation of TEM Specimens from Battery Cathodes. Microsc. Microanal..

[69] Li J., Lu W., Gibson J., Zhang S., Chen T., Korte-Kerzel S., Raabe D. (2018). Eliminating Deformation Incompatibility in Composites by Gradient Nanolayer Architectures. Sci. Rep..

[70] Yun S.-H., Yoo S.M., Sohn B.-H., Jung J.C., Zin W.-C., Kwak S.-Y., Lee T.S. (2005). Electrically Anisotropic Thin Films Consisting of Polymeric and Metallic Nanolayers from Self-Assembled Lamellae of Diblock Copolymers. Langmuir.

[71] Kim J.-Y., Kim J.-I., Seo W.-S. (2011). Exfoliation Route to Nanostructured Cobalt Oxide with Enhanced Thermoelectric Performance. Appl. Phys. Express.

[72] Montana J.-S., Roland S., Richaud E., Miquelard-Garnier G. (2018). From Equilibrium Lamellae to Out-of-Equilibrium Cylinders in Triblock Copolymer Nanolayers Obtained via Multilayer Coextrusion. Polymer.

[73] De Samber B., De Rycke R., De Bruyne M., Kienhuis M., Sandblad L., Bohic S., Cloetens P., Urban C., Polerecky L., Vincze L. (2020). Effect of Sample Preparation Techniques upon Single Cell Chemical Imaging: A Practical Comparison between Synchrotron Radiation Based X-Ray Fluorescence (SR-XRF) and Nanoscopic Secondary Ion Mass Spectrometry (Nano-SIMS). Anal. Chim. Acta.

[74] Timmermans N., Van Meer M., Okhuijsen R., Chen Q. (2024). Process Optimization of Broad Ion Beam Milling for Preparation of Coating Cross-Sections. Ultramicroscopy.

[75] Sharma P., Maya P.N., Satyaprasad A., Deshpande S.P. (2024). Characterization of Vacancy Defects Using TEM in Heavy-Ion-Irradiated Tungsten Foils. Met. Mater. Trans. A.

[76] Dieterle L., Butz B., Müller E. (2011). Optimized Ar+-Ion Milling Procedure for TEM Cross-Section Sample Preparation. Ultramicroscopy.

[77] Santos A.J., Lacroix B., Maudet F., Paumier F., Hurand S., Dupeyrat C., Gómez V.J., Huffaker D.L., Girardeau T., García R. (2022). Application of Advanced (S)TEM Methods for the Study of Nanostructured Porous Functional Surfaces: A Few Working Examples. Mater. Charact..

[78] Kant K., Losic D., Wang Z.M. (2013). Focused Ion Beam (FIB) Technology for Micro- and Nanoscale Fabrications. FIB Nanostructures.

[79] Practical Electron Microscopy and Database—An Online Book. https://www.globalsino.com/EM/.

[80] Hofmann F., Harder R.J., Liu W., Liu Y., Robinson I.K., Zayachuk Y. (2018). Glancing-Incidence Focussed Ion Beam Milling: A Coherent X-Ray Diffraction Study of 3D Nano-Scale Lattice Strains and Crystal Defects. Acta Mater..

[81] Huang Z. (2004). Combining Ar Ion Milling with FIB Lift-out Techniques to Prepare High Quality Site-specific TEM Samples. J. Microsc..

[82] Singh D.R.P., Chawla N., Shen Y.-L. (2010). Focused Ion Beam (FIB) Tomography of Nanoindentation Damage in Nanoscale Metal/Ceramic Multilayers. Mater. Charact..

[83] Lévy D., Aléon J., Aléon-Toppani A., Troadec D., Duhamel R., Gonzalez-Cano A., Bureau H., Khodja H. (2019). NanoSIMS Imaging of D/H Ratios on FIB Sections. Anal. Chem..

[84] Mitchell S. Which Method of Sectioning Is Best for My Sample?. https://www.eag.com/app-note/which-method-of-sectioning-is-best-for-my-sample/.

[85] Huang L., Gan Y. (2024). A Review on SEM Imaging of Graphene Layers. Micron.

[86] Suga M., Asahina S., Sakuda Y., Kazumori H., Nishiyama H., Nokuo T., Alfredsson V., Kjellman T., Stevens S.M., Cho H.S. (2014). Recent Progress in Scanning Electron Microscopy for the Characterization of Fine Structural Details of Nano Materials. Prog. Solid State Chem..

[87] Akhtar K., Khan S.A., Khan S.B., Asiri A.M., Sharma S.K. (2018). Scanning Electron Microscopy: Principle and Applications in Nanomaterials Characterization. Handbook of Materials Characterization.

[88] Bernstein G.H., Carter A.D., Joy D.C. (2013). Do SE_II_ Electrons Really Degrade SEM Image Quality?. Scanning.

[89] Jiang Q., Rogez B., Claude J.-B., Moreau A., Lumeau J., Baffou G., Wenger J. (2020). Adhesion Layer Influence on Controlling the Local Temperature in Plasmonic Gold Nanoholes. Nanoscale.

[90] Young S., Weston D., Dauksher B., Mancini D., Pacheco S., Zurcher P., Miller M. (2005). A Novel Low-Temperature Method to Fabricate MEMS Resonators Using PMGI as a Sacrificial Layer. J. Micromech. Microeng..

[91] Egerton R.F. (2016). Physical Principles of Electron Microscopy.

[92] Dwyer C. (2021). Quantitative Annular Dark-Field Imaging in the Scanning Transmission Electron Microscope—A Review. J. Phys. Mater..

[93] MacLaren I., Fraser A.T., Lipsett M.R., Ophus C. (2024). Digital Dark Field—Higher Contrast and Greater Specificity Dark Field Imaging Using a 4DSTEM Approach. Microsc. Microanal..

[94] Luo R., Gao M., Wang C., Zhu J., Guzman R., Zhou W. (2024). Probing Functional Structures, Defects, and Interfaces of 2D Transition Metal Dichalcogenides by Electron Microscopy. Adv. Funct. Mater..

[95] Mayeen A., Shaji L.K., Nair A.K., Kalarikkal N. (2018). Morphological Characterization of Nanomaterials. Characterization of Nanomaterials.

[96] Klenov D., Freitag B., Von Harrach H., D’Alfonso A., Allen L. (2011). Chemical Mapping at the Atomic Level Using Energy Dispersive X-Ray Spectroscopy. Microsc. Microanal..

[97] Wang X., Yuan X., Gong D., Cheng X., Li K. (2021). Optical Properties and Thermal Stability of AlCrON-Based Multilayer Solar Selective Absorbing Coating for High Temperature Applications. J. Mater. Res. Technol..

[98] Newbury D.E., Ritchie N.W.M. (2015). Performing Elemental Microanalysis with High Accuracy and High Precision by Scanning Electron Microscopy/Silicon Drift Detector Energy-Dispersive X-Ray Spectrometry (SEM/SDD-EDS). J. Mater. Sci..

[99] Von Harrach H., Klenov D., Freitag B., Schlossmacher P., Collins P., Fraser H. (2010). Comparison of the Detection Limits of EDS and EELS in S/TEM. Microsc. Microanal..

[100] Bosman M., Watanabe M., Alexander D.T.L., Keast V.J. (2006). Mapping Chemical and Bonding Information Using Multivariate Analysis of Electron Energy-Loss Spectrum Images. Ultramicroscopy.

[101] Utsumi J., Ide K., Ichiyanagi Y. (2019). Cu/SiO2 Hybrid Bonding Obtained by Surface-Activated Bonding Method at Room Temperature Using Si Ultrathin Films. Micro Nano Eng..

[102] McMahon W.E., Melamed C.L., Tamboli A.E., Toberer E.S., Norman A.G. (2020). Growth of GaAs on Single-Crystal Layered-2D Bi2Se3. J. Cryst. Growth.

[103] Lv J., Zhang H., Zhang D., Liu L., Han Y. (2022). Low-Dose Electron Microscopy Imaging of Electron Beam-Sensitive Crystalline Materials. Acc. Mater. Res..

[104] Brodusch N., Gauvin R. (2024). Low Voltage Scanning Transmission Electron Microscopy as a Viable Tool for Routine Analysis of Materials Science Specimens. Microsc. Microanal..

[105] Nordström R., Zhu L., Härmark J., Levi-Kalisman Y., Koren E., Barenholz Y., Levinton G., Shamrakov D. (2021). Quantitative Cryo-TEM Reveals New Structural Details of Doxil-Like PEGylated Liposomal Doxorubicin Formulation. Pharmaceutics.

[106] Wang D., Russell T.P. (2018). Advances in Atomic Force Microscopy for Probing Polymer Structure and Properties. Macromolecules.

[107] Collinson D.W., Sheridan R.J., Palmeri M.J., Brinson L.C. (2021). Best Practices and Recommendations for Accurate Nanomechanical Characterization of Heterogeneous Polymer Systems with Atomic Force Microscopy. Prog. Polym. Sci..

[108] Bian K., Gerber C., Heinrich A.J., Müller D.J., Scheuring S., Jiang Y. (2021). Scanning Probe Microscopy. Nat. Rev. Methods Primers.

[109] Dazzi A., Prazeres R., Glotin F., Ortega J.M. (2005). Local Infrared Microspectroscopy with Subwavelength Spatial Resolution with an Atomic Force Microscope Tip Used as a Photothermal Sensor. Opt. Lett..

[110] Hoogenboom B.W. (2021). Stretching the Resolution Limit of Atomic Force Microscopy. Nat. Struct. Mol. Biol..

[111] Xie Q., Xu X.G. (2023). What Do Different Modes of AFM-IR Mean for Measuring Soft Matter Surfaces?. Langmuir.

[112] Robson A.J., Grishin I., Young R.J., Sanchez A.M., Kolosov O.V., Hayne M. (2013). High-Accuracy Analysis of Nanoscale Semiconductor Layers Using Beam-Exit Ar-Ion Polishing and Scanning Probe Microscopy. ACS Appl. Mater. Interfaces.

[113] Benaglia S., Amo C.A., Garcia R. (2019). Fast, Quantitative and High Resolution Mapping of Viscoelastic Properties with Bimodal AFM. Nanoscale.

[114] Dazzi A., Prater C.B. (2017). AFM-IR: Technology and Applications in Nanoscale Infrared Spectroscopy and Chemical Imaging. Chem. Rev..

[115] Yang C., Dang C.-Q., Zhu W.-L., Ju B.-F. (2023). High-Speed Atomic Force Microscopy in Ultra-Precision Surface Machining and Measurement: Challenges, Solutions and Opportunities. Surf. Sci. Technol..

[116] Petrov Y.V., Vyvenko O.F., Hlawacek G., Gölzhäuser A. (2016). Secondary Electron Generation in the Helium Ion Microscope: Basics and Imaging. Helium Ion Microscopy.

[117] Joy D.C. (2013). Helium Ion Microscopy: Principles and Applications.

[118] Wirtz T., De Castro O., Audinot J.-N., Philipp P. (2019). Imaging and Analytics on the Helium Ion Microscope. Annu. Rev. Anal. Chem..

[119] Boden S.A., Franklin T.M.W., Scipioni L., Bagnall D.M., Rutt H.N. (2012). Ionoluminescence in the Helium Ion Microscope. Microsc. Microanal..

[120] Veligura V., Hlawacek G., Van Gastel R., Zandvliet H.J.W., Poelsema B. (2012). Channeling in Helium Ion Microscopy: Mapping of Crystal Orientation. Beilstein J. Nanotechnol..

[121] Klingner N., Heller R., Hlawacek G., Facsko S., Von Borany J. (2019). Time-of-Flight Secondary Ion Mass Spectrometry in the Helium Ion Microscope. Ultramicroscopy.

[122] Giurlani W., Berretti E., Innocenti M., Lavacchi A. (2020). Measuring the Thickness of Metal Coatings: A Review of the Methods. Coatings.

[123] Masters R.C., Wan Q., Zhang Y., Dapor M., Sandu A.M., Jiao C., Zhou Y., Zhang H., Lidzey D.G., Rodenburg C. (2017). Novel Organic Photovoltaic Polymer Blends: A Rapid, 3-Dimensional Morphology Analysis Using Backscattered Electron Imaging in the Scanning Electron Microscope. Sol. Energy Mater. Sol. Cells.

[124] Spiliopoulos P., Gestranius M., Zhang C., Ghiyasi R., Tomko J., Arstila K., Putkonen M., Hopkins P.E., Karppinen M., Tammelin T. (2022). Cellulose-Inorganic Hybrids of Strongly Reduced Thermal Conductivity. Cellulose.

[125] Terpstra A.S., Shopsowitz K.E., Gregory C.F., Manning A.P., Michal C.A., Hamad W.Y., Yang J., MacLachlan M.J. (2013). Helium Ion Microscopy: A New Tool for Imaging Novel Mesoporous Silica and Organosilica Materials. Chem. Commun..

[126] Gunawardane R.P., Arumainayagam C.R., Vij D.R. (2006). Auger Electron Spectroscopy. Handbook of Applied Solid State Spectroscopy.

[127] Martinez E., Yadav P., Bouttemy M., Renault O., Borowik Ł., Bertin F., Etcheberry A., Chabli A. (2013). Scanning Auger Microscopy for High Lateral and Depth Elemental Sensitivity. J. Electron. Spectrosc. Relat. Phenom..

[128] Robinson K.J., Thissen H. (2024). Selecting the Best Surface Analysis Method for Your Materials/Samples. J. Vac. Sci. Technol. A.

[129] De Hosson J.T.M., Carvalho N.J.M., Pei Y., Galvan D., Cavaleiro A., De Hosson J.T.M. (2006). Electron Microscopy Characterization of Nanostructured Coatings. Nanostructured Coatings.

[130] Fu J., Fan Z., Nakabayashi M., Ju H., Pastukhova N., Xiao Y., Feng C., Shibata N., Domen K., Li Y. (2022). Interface Engineering of Ta3N5 Thin Film Photoanode for Highly Efficient Photoelectrochemical Water Splitting. Nat. Commun..

[131] Weber P.K., Debliqui M., Defouilloy C., Mayali X., Liu M.-C., Hestrin R., Pett-Ridge J., Stuart R., Morris M., Ramon C. (2024). The NanoSIMS-HR: The Next Generation of High Spatial Resolution Dynamic SIMS. Anal. Chem..

[132] Sangely L., Boyer B., De Chambost E., Valle N., Audinot J.-N., Ireland T., Wiedenbeck M., Aléon J., Jungnickel H., Barnes J.-P., Prohaska T., Irrgeher J., Zitek A., Jakubowski N. (2014). Secondary Ion Mass Spectrometry. Sector Field Mass Spectrometry for Elemental and Isotopic Analysis.

[133] Nuñez J., Renslow R., Cliff J.B., Anderton C.R. (2018). NanoSIMS for Biological Applications: Current Practices and Analyses. Biointerphases.

[134] Wacey D., Kilburn M.R., Mcloughlin N., Parnell J., Stoakes C.A., Grovenor C.R.M., Brasier M.D. (2008). Use of NanoSIMS in the Search for Early Life on Earth: Ambient Inclusion Trails in a *c*. 3400 Ma Sandstone. J. Geol. Soc..

[135] Wang Y., Schreiber D.K., Neeway J.J., Thevuthasan S., Evans J.E., Ryan J.V., Zhu Z., Wei W.D. (2014). NanoSIMS Imaging Alteration Layers of a Leached SON68 Glass via a FIB-made Wedged Crater. Surf. Interface Anal..

[136] Otter L.M., Eder K., Kilburn M.R., Yang L., O’Reilly P., Nowak D.B., Cairney J.M., Jacob D.E. (2023). Growth Dynamics and Amorphous-to-Crystalline Phase Transformation in Natural Nacre. Nat. Commun..

[137] Höppener C., Schacher F.H., Deckert V. (2020). Multimodal Characterization of Resin Embedded and Sliced Polymer Nanoparticles by Means of Tip-Enhanced Raman Spectroscopy and Force–Distance Curve Based Atomic Force Microscopy. Small.

[138] Kumar N., Mignuzzi S., Su W., Roy D. (2015). Tip-Enhanced Raman Spectroscopy: Principles and Applications. EPJ Tech. Instrum..

[139] Sacco A., Imbraguglio D., Giovannozzi A.M., Portesi C., Rossi A.M. (2018). Development of a Candidate Reference Sample for the Characterization of Tip-Enhanced Raman Spectroscopy Spatial Resolution. RSC Adv..

[140] Zhang R., Zhang Y., Dong Z.C., Jiang S., Zhang C., Chen L.G., Zhang L., Liao Y., Aizpurua J., Luo Y. (2013). Chemical Mapping of a Single Molecule by Plasmon-Enhanced Raman Scattering. Nature.

[141] Purushothaman S.M., Tronco M.F., Ponçot M., C.S C., Guigo N., Malfois M., Kalarikkal N., Thomas S., Royaud I., Rouxel D. (2024). Quantifying the Crystalline Polymorphism in PVDF: Comparative Criteria Using DSC, WAXS, FT-IR, and Raman Spectroscopy. ACS Appl. Polym. Mater..

[142] Chen C., Hayazawa N., Kawata S. (2014). A 1.7 Nm Resolution Chemical Analysis of Carbon Nanotubes by Tip-Enhanced Raman Imaging in the Ambient. Nat. Commun..

[143] Hitchcock A.P. (2015). Soft X-Ray Spectromicroscopy and Ptychography. J. Electron. Spectrosc. Relat. Phenom..

[144] Andrews J.C., Meirer F., Liu Y., Mester Z., Pianetta P. (2011). Transmission X-ray Microscopy for Full-field Nano Imaging of Biomaterials. Microsc. Res. Tech..

[145] Watts B., McNeill C.R., Raabe J. (2012). Imaging Nanostructures in Organic Semiconductor Films with Scanning Transmission X-Ray Spectro-Microscopy. Synth. Met..

[146] Scholl A. (2003). Applications of Photoemission Electron Microscopy (PEEM) in Magnetism Research. Curr. Opin. Solid State Mater. Sci..

[147] Barinov A., Dudin P., Gregoratti L., Locatelli A., Onur Menteş T., Ángel Niño M., Kiskinova M. (2009). Synchrotron-Based Photoelectron Microscopy. Nucl. Instrum. Methods Phys. Res. Sect. A Accel. Spectrometers Detect. Assoc. Equip..

[148] Watts B., Thomsen L., Dastoor P.C. (2006). Methods in Carbon K-Edge NEXAFS: Experiment and Analysis. J. Electron. Spectrosc. Relat. Phenom..

[149] Hieulle J. (2014). Structures and Electronic Properties of Organic Self-Assembled Monolayers Characterized by STM and XPS. Ph.D. Thesis.

[150] Newville M. (2004). Fundamentals of XAFS.

[151] Watts B., Schuettfort T., McNeill C.R. (2011). Mapping of Domain Orientation and Molecular Order in Polycrystalline Semiconducting Polymer Films with Soft X-Ray Microscopy. Adv. Funct. Mater..

[152] Foetisch A., Filella M., Watts B., Vinot L.-H., Bigalke M. (2022). Identification and Characterisation of Individual Nanoplastics by Scanning Transmission X-Ray Microscopy (STXM). J. Hazard. Mater..

[153] Urquhart S.G. (2022). X-Ray Spectroptychography. ACS Omega.

[154] Hitchcock A.P., Zhang C., Eraky H., Higgins D., Belkhou R., Millle N., Swaraj S., Stanescu S., Sun T., Wang J. (2024). Comparison of Soft X-Ray Spectro-Ptychography and Scanning Transmission X-Ray Microscopy. J. Electron. Spectrosc. Relat. Phenom..

[155] Guttmann P., Bittencourt C., Rehbein S., Umek P., Ke X., Van Tendeloo G., Ewels C.P., Schneider G. (2012). Nanoscale Spectroscopy with Polarized X-Rays by NEXAFS-TXM. Nat. Photonics.

[156] Xie T., Bai L., Liu J., Zhao G., Sun X., Zhong J. (2013). X-Ray Induced Carbon Coating on Carbon Nanotubes. Carbon.

[157] De Jesus L.R., Dennis R.V., Depner S.W., Jaye C., Fischer D.A., Banerjee S. (2013). Inside and Outside: X-Ray Absorption Spectroscopy Mapping of Chemical Domains in Graphene Oxide. J. Phys. Chem. Lett..

[158] Atom Probe Tomography—Introduction to the Technique n.d. https://www.cameca.com/products/apt/technique.

[159] Miller M.K., Kelly Thomas F., Rajan K., Ringer S.P. (2012). The future of atom probe tomography. Mater. Today.

[160] Gault B., Chiaramonti A., Cojocaru-Mirédin O., Stender P., Dubosq R., Freysoldt C., Makineni S.K., Li T., Moody M., Cairney J.M. (2021). Atom probe tomography. Nat. Rev. Methods Primers.

[161] Thompson K., Lawrence D., Larson D.J., Olson J.D., Kelly T.F., Gorman B. (2007). In situ site-specific specimen preparation for atom probe tomography. Ultramicroscopy.

[162] Yang T., Zhao Y.L., Li W.P., Yu C.Y., Luan J.H., Lin D.Y., Fan L., Jiao Z.B., Liu W.H., Liu X.J. (2020). Ultrahigh-Strength and Ductile Superlattice Alloys with Nanoscale Disordered Interfaces. Science.

[163] Brognara A., Jung C., Poltronieri C., Djemia P., Dehm G., Ghidelli M., Best J.P. (2024). Thermal Modification of ZrCu Metallic Glass Nanolaminates: Structure and Mechanical Properties. arXiv.

[164] Weyland M., Midgley P.A. (2004). Electron Tomography. Mater. Today.

[165] Ersen O., Hirlimann C., Drillon M., Werckmann J., Tihay F., Pham-Huu C., Crucifix C., Schultz P. (2007). 3D-TEM Characterization of Nanometric Objects. Solid State Sci..

[166] Ercius P., Alaidi O., Rames M.J., Ren G. (2015). Electron Tomography: A Three-Dimensional Analytic Tool for Hard and Soft Materials Research. Adv. Mater..

[167] Chang S., Li L., Hong B., Liu J., Xu Y., Pang K., Zhang L., Han H., Chen X. (2023). An Intelligent Workflow for Sub-Nanoscale 3D Reconstruction of Intact Synapses from Serial Section Electron Tomography. BMC Biol..

[168] Bals S., Van Tendeloo G., Kisielowski C. (2006). A New Approach for Electron Tomography: Annular Dark-Field Transmission Electron Microscopy. Adv. Mater..

[169] Hata S., Furukawa H., Gondo T., Hirakami D., Horii N., Ikeda K.-I., Kawamoto K., Kimura K., Matsumura S., Mitsuhara M. (2020). Electron Tomography Imaging Methods with Diffraction Contrast for Materials Research. Microscopy.

[170] Haberfehlner G., Orthacker A., Albu M., Li J., Kothleitner G. (2014). Nanoscale Voxel Spectroscopy by Simultaneous EELS and EDS Tomography. Nanoscale.

[171] Collins S.M., Midgley P.A. (2017). Progress and Opportunities in EELS and EDS Tomography. Ultramicroscopy.

[172] Koneti S., Roiban L., Dalmas F., Langlois C., Gay A.-S., Cabiac A., Grenier T., Banjak H., Maxim V., Epicier T. (2019). Fast Electron Tomography: Applications to Beam Sensitive Samples and in Situ TEM or Operando Environmental TEM Studies. Mater. Charact..

[173] Xia W., Yang Y., Meng Q., Deng Z., Gong M., Wang J., Wang D., Zhu Y., Sun L., Xu F. (2018). Bimetallic Nanoparticle Oxidation in Three Dimensions by Chemically Sensitive Electron Tomography and in Situ Transmission Electron Microscopy. ACS Nano.

[174] Eijsbouts S., Li X., Juan-Alcaniz J., Van Den Oetelaar L.C.A., Bergwerff J.A., Loos J., Carlsson A., Vogt E.T.C. (2017). Electron Tomography Reveals the Active Phase–Support Interaction in Sulfidic Hydroprocessing Catalysts. ACS Catal..

[175] Holler M., Diaz A., Guizar-Sicairos M., Karvinen P., Färm E., Härkönen E., Ritala M., Menzel A., Raabe J., Bunk O. (2014). X-Ray Ptychographic Computed Tomography at 16 Nm Isotropic 3D Resolution. Sci. Rep..

[176] Kahnt M., Sala S., Johansson U., Björling A., Jiang Z., Kalbfleisch S., Lenrick F., Pikul J.H., Thånell K. (2020). First Ptychographic X-Ray Computed Tomography Experiment on the NanoMAX Beamline. J. Appl. Crystallogr..

[177] Górecki R., Polo C.C., Kalile T.A., Miqueles E.X.S., Tonin Y.R., Upadhyaya L., Meneau F., Nunes S.P. (2023). Ptychographic X-Ray Computed Tomography of Porous Membranes with Nanoscale Resolution. Commun. Mater..

[178] Pedersen E.B.L., Angmo D., Dam H.F., Thydén K.T.S., Andersen T.R., Skjønsfjell E.T.B., Krebs F.C., Holler M., Diaz A., Guizar-Sicairos M. (2015). Improving Organic Tandem Solar Cells Based on Water-Processed Nanoparticles by Quantitative 3D Nanoimaging. Nanoscale.

[179] Yuan H., Yuan H., Casagrande T., Shapiro D., Yu Y.-S., Enders B., Lee J.R.I., Van Buuren A., Biener M.M., Gammon S.A. (2021). 4D Imaging of ZnO-Coated Nanoporous Al_2_ O_3_ Aerogels by Chemically Sensitive Ptychographic Tomography: Implications for Designer Catalysts. ACS Appl. Nano Mater..

[180] Ponomaryov S.S., Yukhymchuk V.O., Lytvyn P.M., Valakh M.Y. (2016). Direct Determination of 3D Distribution of Elemental Composition in Single Semiconductor Nanoislands by Scanning Auger Microscopy. Nanoscale Res. Lett..

[181] Li K., Liu J., Grovenor C.R.M., Moore K.L. (2020). NanoSIMS Imaging and Analysis in Materials Science. Annu. Rev. Anal. Chem..

[182] Michelson A., Minevich B., Emamy H., Huang X., Chu Y.S., Yan H., Gang O. (2022). Three-Dimensional Visualization of Nanoparticle Lattices and Multimaterial Frameworks. Science.

[183] Aidukas T., Phillips N.W., Diaz A., Poghosyan E., Müller E., Levi A.F.J., Aeppli G., Guizar-Sicairos M., Holler M. (2024). High-Performance 4-Nm-Resolution X-Ray Tomography Using Burst Ptychography. Nature.

[184] Mille N., Yuan H., Vijayakumar J., Stanescu S., Swaraj S., Desjardins K., Favre-Nicolin V., Belkhou R., Hitchcock A.P. (2022). Ptychography at the Carbon K-Edge. Commun. Mater..

[185] Mathurin J., Deniset-Besseau A., Bazin D., Dartois E., Wagner M., Dazzi A. (2022). Photothermal AFM-IR Spectroscopy and Imaging: Status, Challenges, and Trends. J. Appl. Phys..

[186] Wieland K., Ramer G., Weiss V.U., Allmaier G., Lendl B., Centrone A. (2019). Nanoscale Chemical Imaging of Individual Chemotherapeutic Cytarabine-Loaded Liposomal Nanocarriers. Nano Res..

[187] dos Santos A.C.V.D., Hondl N., Ramos-Garcia V., Kuligowski J., Lendl B., Ramer G. (2023). AFM-IR for Nanoscale Chemical Characterization in Life Sciences: Recent Developments and Future Directions. ACS Meas. Sci. Au.

[188] Dazzi A., Mathurin J., Leclere P., Nickmilder P., De Wolf P., Wagner M., Hu Q., Deniset-Besseau A. (2024). Photothermal AFM-IR Depth Sensitivity: An Original Pathway to Tomographic Reconstruction. Anal. Chem..

[189] Jakob D.S., Wang H., Zeng G., Otzen D.E., Yan Y., Xu X.G. (2020). Peak Force Infrared–Kelvin Probe Force Microscopy. Angew. Chem. Int. Ed..

[190] Rutherford D., Kolářová K., Čech J., Haušild P., Kuliček J., Ukraintsev E., Stehlík Š., Dao R., Neuman J., Rezek B. (2024). Correlative Atomic Force Microscopy and Scanning Electron Microscopy of Bacteria-Diamond-Metal Nanocomposites. Ultramicroscopy.

[191] Hughes L.A., Savitzky B.H., Deng H.D., Jin N.L., Lomeli E.G., Yu Y.-S., Shapiro D.A., Herring P., Anapolsky A., Chueh W.C. (2022). Correlative Analysis of Structure and Chemistry of LixFePO4 Platelets Using 4D-STEM and X-Ray Ptychography. Mater. Today.

[192] Jia H., Wang C., Wang C., Clancy P. (2023). Machine Learning Approach to Enable Spectral Imaging Analysis for Particularly Complex Nanomaterial Systems. ACS Nano.

[193] Aoyagi S. (2023). Application of Machine Learning to Spectrum and Image Data. J. Vac. Sci. Technol. A.

